# A New Species of the Bay Goby Genus *Eucyclogobius*, Endemic to Southern California: Evolution, Conservation, and Decline

**DOI:** 10.1371/journal.pone.0158543

**Published:** 2016-07-27

**Authors:** Camm C. Swift, Brenton Spies, Ryan A. Ellingson, David K. Jacobs

**Affiliations:** 1 Emeritus, Natural History Museum of Los Angeles County, 900 Exposition Boulevard, Los Angeles, California 90007, United States of America; 2 Department of Ecology and Evolutionary Biology, University of California, Los Angeles, California 90095, United States of America; 3 Department of Biological Sciences, California State University, Los Angeles, California 90032, United States of America; National Cheng-Kung University, TAIWAN

## Abstract

A geographically isolated set of southern localities of the formerly monotypic goby genus *Eucyclogobius* is known to be reciprocally monophyletic and substantially divergent in mitochondrial sequence and nuclear microsatellite-based phylogenies relative to populations to the north along the California coast. To clarify taxonomic and conservation status, we conducted a suite of analyses on a comprehensive set of morphological counts and measures from across the range of *Eucyclogobius* and describe the southern populations as a new species, the Southern Tidewater Goby, *Eucyclogobius kristinae*, now separate from the Northern Tidewater Goby *Eucyclogobius newberryi* (Girard 1856). In addition to molecular distinction, adults of *E*. *kristinae* are diagnosed by: 1) loss of the anterior supratemporal lateral-line canals resulting in higher neuromast counts, 2) lower pectoral and branched caudal ray counts, and 3) sets of measurements identified via discriminant analysis. These differences suggest ecological distinction of the two species. Previous studies estimated lineage separation at 2–4 million years ago, and mitochondrial sequence divergence exceeds that of other recognized fish species. Fish from Santa Monica Artesian Springs (Los Angeles County) northward belong to *E*. *newberryi*; those from Aliso Creek (Orange County) southward constitute *E*. *kristinae*. The lagoonal habitat of *Eucyclogobius* has been diminished or degraded, leading to special conservation status at state and federal levels beginning in 1980. Habitat of the newly described species has been impacted by a range of anthropogenic activities, including the conversion of closing lagoons to open tidal systems in the name of restoration. In the last 30 years, *E*. *kristinae* has only been observed in nine intermittently occupied lagoonal systems in northern San Diego County; it currently persists in only three sites. Thus, the new species is in imminent danger of extinction and will require ongoing active management.

## Introduction

### General

The monotypic goby genus *Eucyclogobius* Gill, 1862, type *Gobius newberryi* Girard, 1856[1], was described based on specimens from northern California, in or near Walker Creek in Tomales Bay [1–4]. *Eucyclogobius* was known exclusively from central and northern California prior to 1939, the year it was first collected from coastal California south of the Los Angeles plain [5,6]. These small fish inhabit coastal stream-mouth lagoons and bay-margin ponded water habitats of variable, but often low, salinity. In these habitats *Eucyclogobius* exhibits local persistence in isolation in some areas, but local extirpation and recolonization with limited local marine dispersal [7,8] in others. Additional salient features of *Eucyclogobius* include reversal of typical sex roles and sexual dimorphism. The males dig burrows where they guard fertilized eggs. Females engage in competition for males where they conduct colorful display rituals at the entrance of the male-occupied burrow prior to egg laying and fertilization [9]. The range of *Eucyclogobius* extends the length of California from Del Norte to San Diego counties. Gaps in this distribution occur in central and northern California where steep, rocky coasts preclude coastal lagoon formation. Such rocky shores separate distinct phylogeographic clades that are evident in both mitochondrial and microsatellite analyses [10–12]. These clades served as the basis for management units in the U.S. Fish and Wildlife Service Recovery Plan [13,14]. In addition, variable reduction of the sensory canals of the head is known to closely parallel these phylogeographic units [15].

Observation of habitat loss through the 1970s and 80s in the face of coastal development and drought, combined with natural history studies [9,13,14,16] suggesting the possibility of genetic subdivision, led to state listing as a species of concern in 1980 and ultimately to federal listing as an endangered species in 1994 [13]. Subsequent work has confirmed deep genetic subdivision [10–12]. Sites are genetically distinct from one another at very short distances along the coast [17,18,19], making this the most locally genetically subdivided vertebrate on the North American Pacific Coast [12]. Multiple lines of evidence support the role of ecology and closed lagoon habitat preference in the generation of this remarkable local genetic differentiation: 1) Recolonization of formerly uninhabited sites coincides with high winter runoff events [7,8] and occurs over distances that are generally less than 10 km, primarily along sandy coasts. 2) Adults tolerate high salinity levels [16] yet larvae and small juveniles are intolerant of marine salinity [20,21]. 3) Deep genetic divergence occurs across headlands and rocky substrate [10–12,18]. In combination, these factors suggest that larvae remain isolated in closed lagoons or fresher water settings, and that adults disperse over soft-sediment substrates near the shore following flood opening of lagoons to the sea. This very limited mode of intermittent dispersal contributes to genetic differentiation and endangerment. Genetic divergence between southern populations and those to the north is much higher than that among still substantially subdivided units of the northern species. In addition, despite challenges presented by sexual dimorphism, allometric growth, and low degree of morphological differentiation, morphological characters reveal consistent differences in both counted and measured characters. These distinctions and their implications for historical ecology, conservation, and recovery are outlined here.

### Taxonomy and Phylogenetic Placement of the Genus *Eucyclogobius*

The genus *Eucyclogobius* has been recognized as monotypic or included as a subgenus with other genera of North Pacific Bay Gobies of the North American coast in an expanded genus *Lepidogobius*, the oldest available name for these related northeastern Pacific gobies (see synonymy below). They share morphological and molecular characters (synapomorphies) with other genera of North Pacific Bay Gobies in Asia. In combination, these form a North Pacific temperate group of gobies that was initially subdivided by Birdsong et al. [22] into two ecologically distinct informal groupings; “Chasmichthys,” (normally proportioned benthic or demersal gobies) and “Astrabe” (elongate infaunal/interstitial gobies with reduced eyes), based on the northwestern Pacific genera *Chasmichthys* and *Astrabe*. Birdsong et al. [22] supported the overall monophyly of the North Pacific Bay Gobies on the pattern of dorsal fin pterygiophore placement in relation to the vertebrae. Thacker [23,24] provided molecular support for this combined “Chasmichthys + Astrabe” group based on a limited number of taxa. Genera sampled from this group fall within the subfamily Gobionellinae [25–27], family Gobionellidae [23,24], or as gobionelline-like gobiids [28] largely based on DNA sequence data. Ellingson et al. [29] and Ellingson [30] provided a comprehensive multi-locus phylogenetic analysis of this North Pacific group, demonstrating its monophyly and that the clade is subdivided into eastern and western temperate North Pacific sister clades. This demonstrated that the elongate Astrabe morphology present in multiple genera on both coasts is a product of convergent evolution. Ellingson et al. [29] confirmed the placement of *Eucyclogobius* within the eastern Pacific Bay Gobies, and that the genus *Eucyclogobius* is sister to the wide-ranging monotypic East Pacific genus *Clevelandia* as first reported by Dawson et al. [11].

### Prior Phylogeographic and Morphologic Evidence of Species-level Distinction of *Eucyclogobius* in Southern California

Dawson et al. [10] documented 4% mitochondrial sequence divergence of the southernmost clade that historically occupied San Diego and Orange County, California, and inferred a divergence time of 2 to 4 million years ago (Mya). This phylogeny, based on DNA sequence data from the mitochondrial control region (D-loop) and cytochrome b gene, placed northern and southern samples into deeply divergent reciprocally monophyletic clades with high bootstrap support (see Figure 3 in reference [10]). Earl et al. [12] used 24 microsatellite loci to generate a nuclear phylogeography of *Eucyclogobius* and recovered a very similar tree topology to the Dawson et al. [10] mitochondrial tree, with comparable reciprocal monophyly between north and south. The southernmost clade was the most distinct based on branch length, as well as dramatic differentiation of allele repeat number at the microsatellite loci themselves [12]. Earl et al. [12] also highlighted the greater divergence of *E*. *kristinae* relative to other canonical endangered species such as Desert Pupfish. Recent work [31] confirms that named pupfish species are orders of magnitude more recent in their divergence than the *Eucyclogobius* species described here. Ahnelt et al. [15] determined that the southern branch of the *Eucyclogobius* phylogeographic tree was morphologically divergent and diagnostically distinct based on characters associated with reduction of sensory canal features of the head. Thus, deeply divergent nuclear and mitochondrial molecular features as well as morphological distinctions have already been established in the literature. Genetic markers also reveal substantially less variation within *E*. *kristinae* relative to *E*. *newberryi*, suggesting small effective population size and underscoring the importance of recognition and conservation of the geographically restricted southern species described here.

### Challenges for the Morphologic Analysis

The morphologic work presented here, particularly regarding morphometrics as opposed to meristics, has challenging aspects that are of interest. Challenges result from 1) constraints on sample size due to the limited museum material available for study, 2) sexual dimorphism of these small fish, 3) the interaction of size, shape, and sex on different characters (despite the selection of adult fish for the analysis), 4) inter clade and population variation, especially in *E*. *newberryi* (where we have samples across the range and from all distinct clades), and 5) the modest morphologic differences between the species. After exploring a range of approaches, including principal components analysis, to examine the numerous measured characters, we settled on two analytical schemes, each is distinct in approach and purpose: a multivariate discriminate function analysis (DFA), and an appraisal of each character using generalized linear models (GLMs). In combination, they provide a comprehensive assessment of the measured character differences between species. In our view, even in the absence of morphological differences, *E*. *kristinae* would merit species designation due to the nuclear and mitochondrial reciprocal monophyly combined with temporal divergence of the lineages (11–12). Here, we use this combination of tools to identify characters that are distinct and provide the capacity to morphologically diagnose the species, while we also explore the morphologic variation associated with size, sex, and species.

We employed discriminate function analysis (DFA) to identify variables that efficiently discriminate between the two species. In preparation for this analysis, we performed a global transformation of all measured variables that normalized for body size. Thus, this approach was not dependent on any particular measure to determine size (e.g. standard length), effectively dealing with the issue of size in an unbiased way. Our second analytical scheme involved treating each measured character independently in a generalized linear model (GLM). The detailed models for each measurement, as well as visualization of the data, revealed association of each character with standard length, species, and sex as well as their interactions. This approach provided a good understanding of how each measured variable was behaving, and permitted a better understanding of distinctions between species as well as preliminary inferences about ecological differences.

Assessment of statistical significance for both morphometric and meristic differences between species was restricted to *alpha* level (p≤0.001), and no other adjustments for multiple sampling were made. Both classes of variables were inspected to determine whether inter-population variation rendered them imperfectly diagnostic between species.

## Materials and Methods

### Counts and Measurements

**Fig 1:**
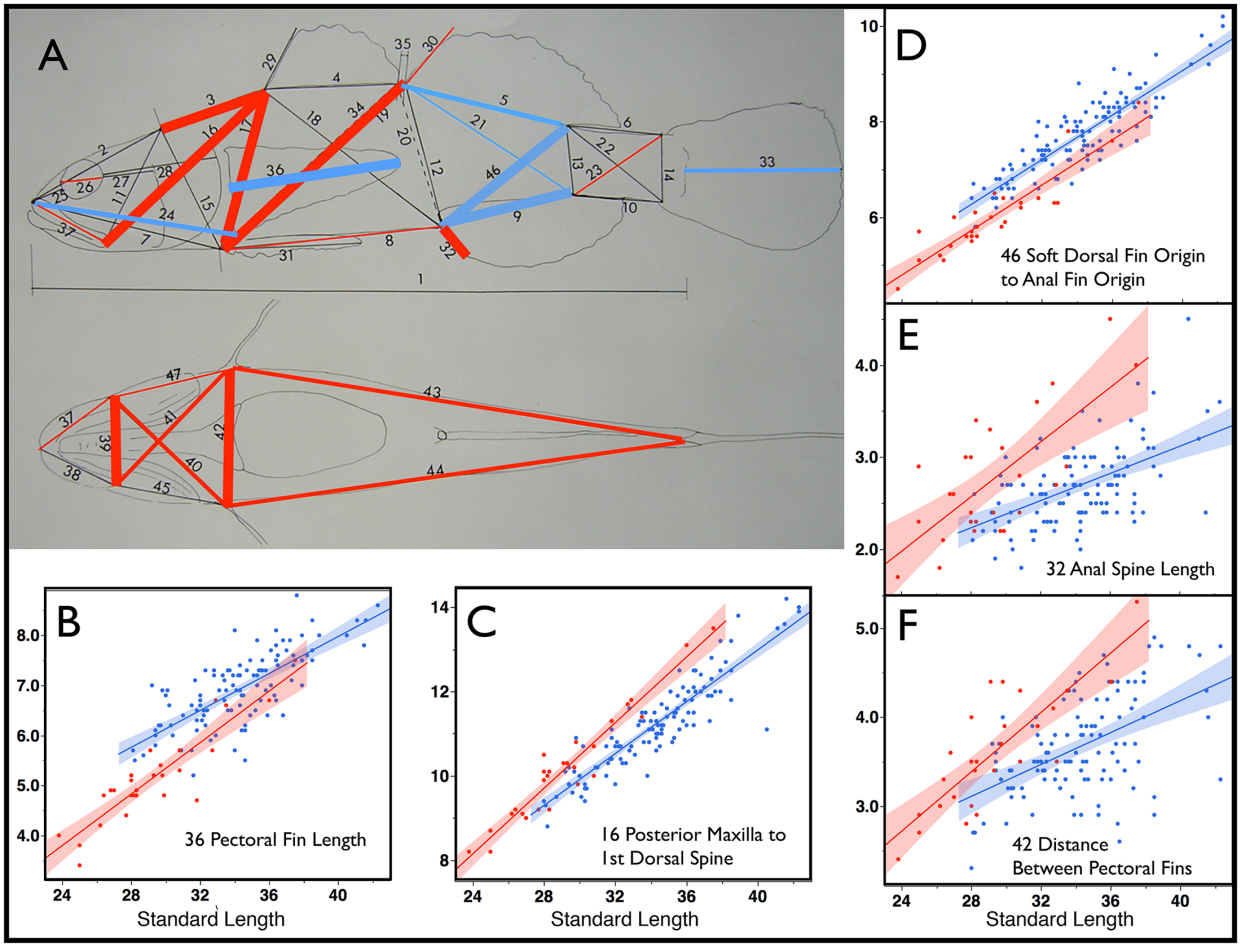
Measured Characters and Species Discrimination. A) Measured characters are numbered on a lateral view (top) and ventral view (bottom), see key below. Colored lines indicate measured characters that statistically differentiate species via a generalized linear model (Table 1). The thickest lines indicate characters that differentiate species at p<0.001, colored lines of intermediate thickness 0.001≤p<0.01, and the thinnest lines indicate 0.01≤p<0.05. Blue lines indicate characters that are larger in *E*. *newberryi*, while red lines indicate characters that are larger in *E*. *kristinae*. B–F graphically present the 5 measures that best differentiate the two species as determined by the discriminant analysis. These variables in combination serve to identify 99% of individuals to species. Colored envelopes around the line form 95% confidence intervals of a least-squares linear model for each species. Data points, regression line, and envelope are in blue for *E*. *newberryi* and in red for *E*. *kristinae*. All measurements are in millimeters. Key to Measured Characters Numbered in Panel A: 1 Standard Length, STANL; 2 Snout tip to occiput (transition of body to head), SNOCC; 3 Occiput to origin (anterior base) of spinous dorsal fin, OCCSP; 4 Length of spinous dorsal fin base, SPNDB; 5 Length of soft dorsal fin base, SFTDB; 6 Soft dorsal fin insertion (posterior end base) to upper caudal fin base, SFTDUCB; 7 Snout tip to pelvic fin origin (to anterior edge of fin), SNOPELO; 8 Pelvic fin origin to anal fin origin, PELOANO; 9 Length of anal fin base, ANALBL; 10 Anal fin insertion (posterior end of fin base) to lower caudal fin origin, ANFILCO; 11 Posterior end maxillary bone to occiput (left side), OCCMAX; 12 Soft dorsal fin origin to anal fin origin, SFTOANO; 13 Soft dorsal insertion to soft anal insertion, SFTDIANI; 14 Depth of caudal peduncle at caudal origin (anterior edge procurrent rays), DEPCAO; 15 Occiput to pelvic origin, OCCPELO; 16 Posterior end of left maxillary bone to spinous (1^st^) dorsal origin, POMXSPDO; 17 Spinous (1^st^) dorsal origin to pelvic fin origin (anterior edge), SPDOPEO; 18 Spinous dorsal fin origin to anal fin origin, SPDOANO; 19 Pelvic fin origin to soft (2^nd^) dorsal fin origin, PELOSFDC; 20 Spinous dorsal fin insertion to anal fin origin, SPDIANO; 21 Soft dorsal fin origin to anal fin insertion, SFTOANI; 22 Soft dorsal fin insertion to ventral caudal fin origin, SFTDIVC; 23 Anal fin insertion to dorsal caudal fin origin, ANIDC; 24 Snout tip to lower pectoral origin, left side, SNOLPECO; 25 Snout tip to anterior fleshy orbit margin, left side, SNORB; 26 Horizontal fleshy orbit diameter, left side, ORBDIA; 27 Posterior margin orbit to posterior edge preopercle, left side, POSTORB; 28 Posterior margin orbit to posterior edge operculum, left side, POSTOPER; 29 Length of first dorsal spine, SPDSPI; 30 Length of first soft dorsal ray or spine, SAFTDRA; 31 Pelvic fin length along mid ventral line, PELVLE; 32 Length of first anal spine or unbranched ray, ANALE; 33 Caudal fin length, CAUDLE; 34 Pelvic fin origin to spinous dorsal fin insertion, PELOSPDI; 35 Between dorsal fins, BETDFINS; 36 Pectoral fin length, left side, PECTLE; 37 Snout to posterior end of left maxillary bone, SNMAXL; 38 Snout to posterior end of right maxillary bone, SNMAXR; 39 Width of mouth at posterior extent, MOUWIDE; 40 Posterior end of left maxillary to right pectoral fin (lower), LMAXRPE; 41 Posterior end right maxillary to left pectoral fin (lower), RMAXLPE; 42 Between lower pectoral fin bases, BETPECO; 43 Lower left pectoral origin to lower caudal fin base, LPECLCAU; 44 Lower right pectoral fin origin (or base) to lower caudal origin, RPECLCAU; 45 Posterior end of right maxillary to lower right pectoral fin, RMAXRPE; 46 Soft Dorsal fin insertion to anal origin, SFDIANO; 47 Posterior end left maxillary bone to lower left pectoral origin, LMAXLPE; 48 (not on figure) Distance between fleshy orbits across top of head, INTORBW.

**Fig 2:**
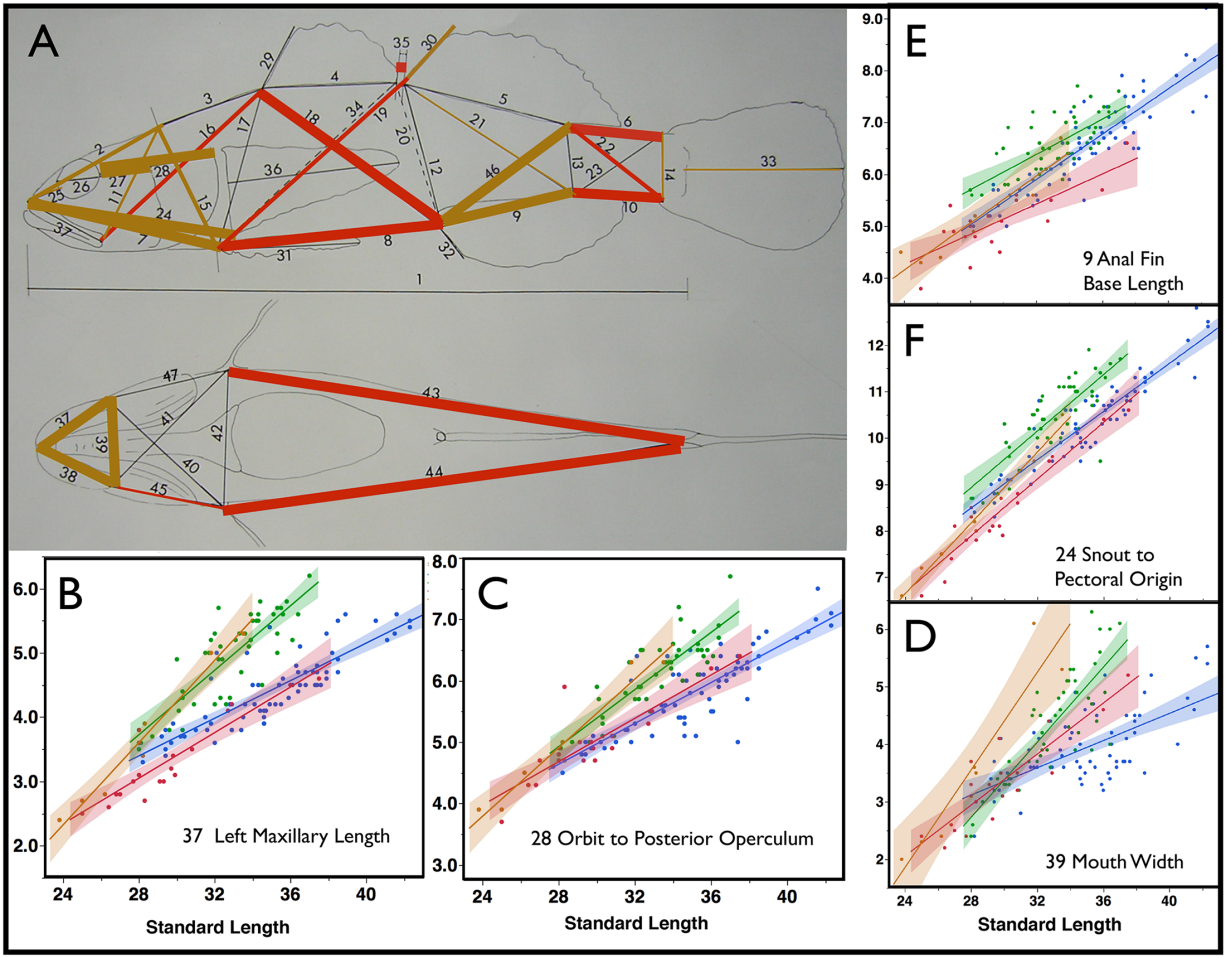
Sexually Dimorphic Characters. In panel A, colored lines indicate measured characters that statistically differentiate sexes in both species *via* a generalized linear model (Table 1). The thickest lines indicate characters that differentiate gender at p<0.001, colored lines of intermediate thickness 0.001≤p<0.01, and the thinnest lines 0.01≤p<0.05. Brown lines indicate characters that are larger in males, while red lines indicate characters that are larger in females. Mandible length and mouth width, as well as some other characters of the head and the anal fin base length are substantially larger in the male than the female. Panels B–D show least squared regression plots for both sexes of each species: *E*. *newberryi* female = blue, male = green; *E*. *kristinae* female = red, male = brown. Mouth characters (B and D) and some head characters (C) are dramatically sexually dimorphic and show allometric differences between males and females as males develop substantially larger mouths toward maturity. E (9 ANALBL) and F (24 SNOLPECO) show characters that are strongly influenced by both sex and species (see table 1); males and *E*. *newberryi* are larger for these characters.

**Fig 3:**
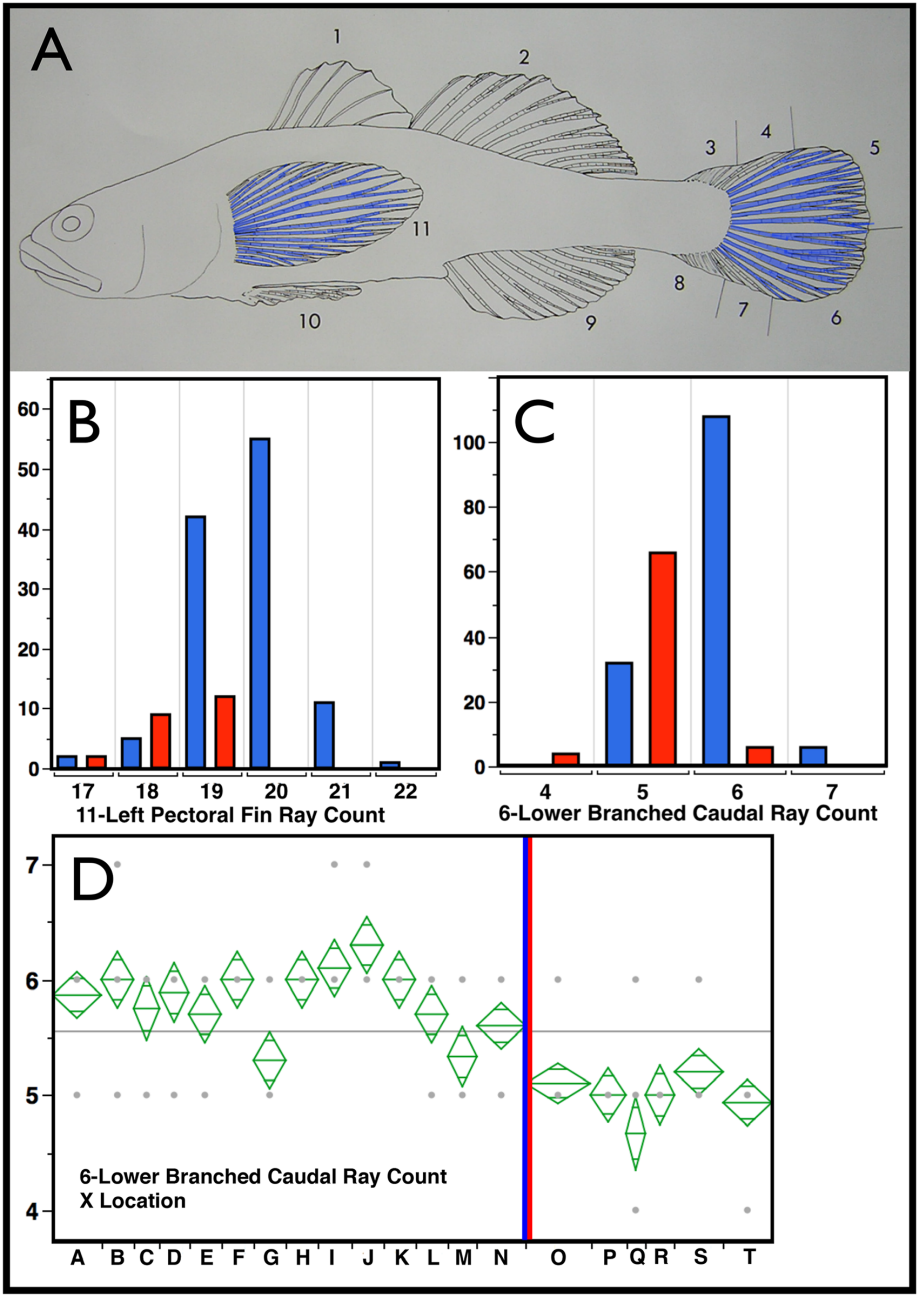
Counts of Fin Rays and Spines. The counted features of the fins are numbered in Panel A, (see key below) and the Left Pectoral Fin Rays (11) as well as the Lower (6) and Upper (5) Branched Caudal Rays are colored blue, signifying that these counts are significantly greater In *E*. *newberryi* than they are in *E*. *kristinae*. Bar graphs in B and C chart the number of individuals of *E*. *newberryi* in blue and *E*. *kristinae* in red that have a particular number of counted rays (x axis) for Pectoral Fin Rays (11) and Lower Branched Caudal Rays (6) respectively. Significance was determined using the non-parametric Wilcoxon Summed Rank Test (implemented in JMP), p<0.0001 in the three cases (well within our *a priori* criteria of p<0.001). Counts related to them and combined with them (Right Pectoral, 11-Left + Right Pectoral; 5-Upper Branched Caudal Rays, Total Branched Caudal Rays (5+6) were also significant at this level. Panel D presents an ANOVA analysis of means for sampling locality for, 6-Lower Branched Caudal Ray count for heuristic comparison. Localities are displayed from north to south with the *E*. *newberryi/E*. *kristinae* geographic split shown by the blue red vertical bar. As can be seen, *E*. *kristinae* by site are consistently lower in this variable. Total branched Caudal Ray Count (5+6 not shown) provides a similarly consistent result. Thus, these counts appear to have modest utility in species diagnosis. Key to localities: A-Smith River, B-Lake Earl, C-Humboldt Bay, D-Brush Creek, E-Estero Americano, F-Rodeo Lagoon, G-Arroyo de Corral, H-Morro Bay†, I-Santa Inez River, J-Jalama Creek, K-Agua Caliente, L-Winchester Canyon†, M-Ventura River, N-Malibu Lagoon†, O-Aliso Creek†, P-San Mateo Creek†, Q-San Onofre Creek, R-Las Pulgas/Las Flores Creek†, S-San Luis Rey River†, T-Buena Vista Lagoon† (most of these habitats are lagoonal in nature, † = currently extirpated, based on absence in recent surveys). Key to fin spine and ray counts: 1-Number of dorsal fin spines, DSPINES; 2-Number of second dorsal fin ray elements, DRAYS; 3-Upper procurrent caudal fin rays, UPRORAY; 4-Upper unbranched primary caudal rays, UUNBRC; 5-Upper branched primary caudal rays, UBRCAR; 6-Lower branched primary caudal rays, LBRCAR; 7-Lower unbranched primary caudal rays, LUNBRCR; 8-Lower procurrent caudal rays, LPRORAY; 9-Number of Anal fin ray elements, ARAYS; 10-Pelvic fin rays, PELELEM; 11-Left pectoral fin rays, LPECRAYS; Right pectoral fin rays, RPECRAYS (not shown); Number of pre-caudal or abdominal vertebrae, PRECAUD (not shown); Number of post-caudal vertebrae (including last half centrum as one), POSTCAUD; (not shown) Total vertebrae, TOTVERT, (not shown). Counts of Fin Rays and Spines are tabulated and averaged by locality in S3 Table.

**Fig 4:**
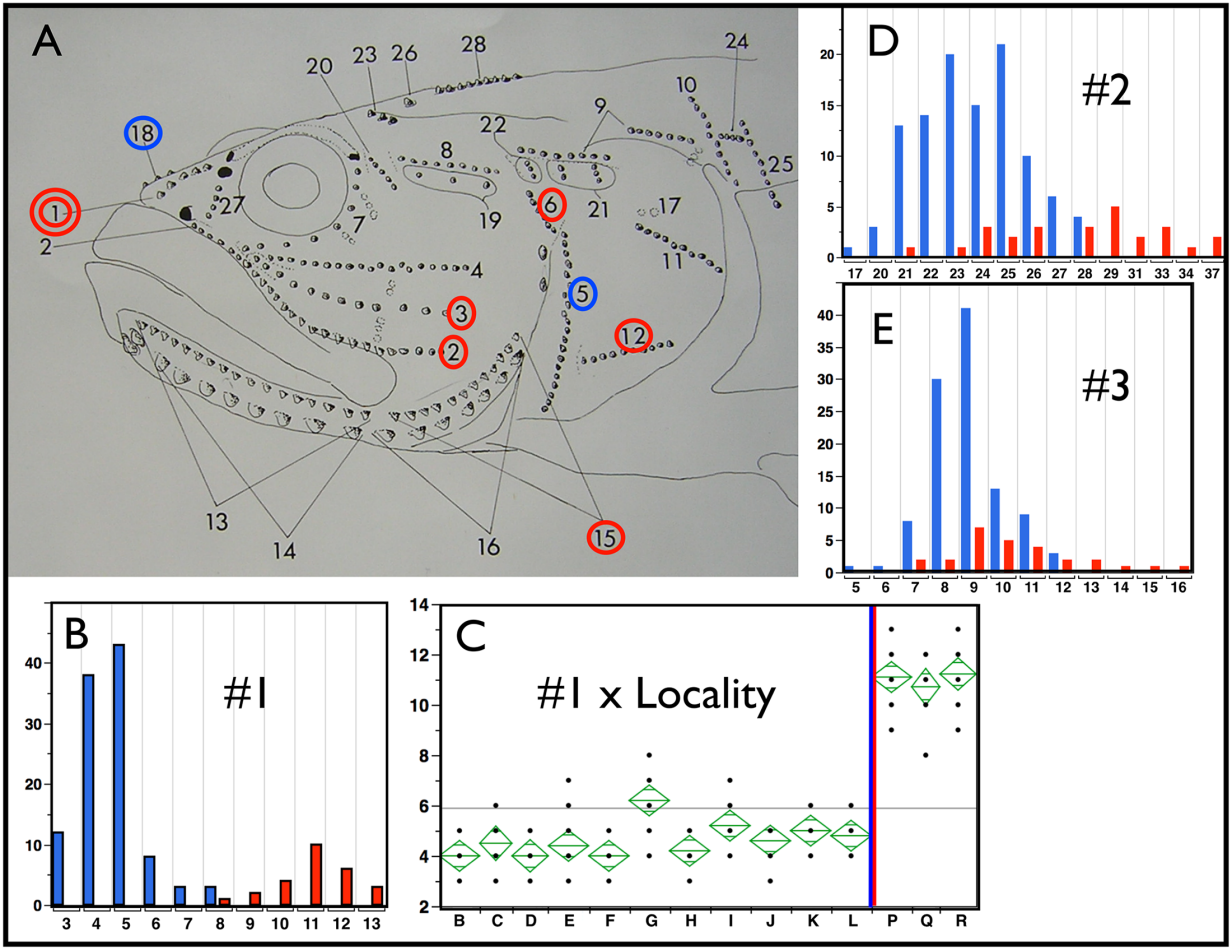
Neuromast Counts. In panel A, circled neuromast counts that are significant at p<0.0001 using the non-parametric Wilcoxon Summed Rank Test (implemented in JMP). Blue circles indicate larger counts for *E*. *newberryi*, red circles larger counts for *E*. *kristinae*. The double circle identifies count #1, which is uniformly diagnostic for all populations and approaches complete diagnosis of individuals. Bar graphs in B, D and E chart the number of individuals of *E*. *newberryi* in blue and *E*. *kristinae* in red that have a particular number of counted neuromasts (x axis) for neuromast counts 1, 2 and 3 respectively. D and E are typical of results that meet our significance criteria but are not necessarily diagnostic at the population or individual level. Panel B, shows a bar graph of count 1 that provides a high level of diagnosis. Panel C presents an ANOVA analysis of means by sampling locality for the neuromast count #1, for heuristic comparison. Localities are displayed from north to south with the *E*. *newberryi/E*. *kristinae* geographic split shown by the blue red vertical bar. Adult fish from these populations are readily identified to species by this count and the absence of the canal over this location (key to localities as in Fig 3 caption). Neuromast Counts are tabulated and averaged by locality in S4 Table.

Forty-seven morphometric measurements (Figs 1 and 2), thirteen fin ray counts (Fig 3), twenty-eight cephalic neuromast line counts (Fig 4), and one count each of gill rakers, vertebrae, and shoulder papillae were analyzed on upwards of 145 specimens from throughout the range of *Eucyclogobius* (see S1 Table for all counts and measures). No animal care protocols were required as all measurements were taken on preserved museum material. Meristic counts were taken on an additional 88 specimens and 23 of the original syntypes of *E*. *newberryi*. Count protocols follow Hubbs and Lagler [32] unless otherwise noted. Counts of the second dorsal and anal fins include the first flexible unsegmented and unbranched spine as well as the following segmented and branched rays. We counted procurrent caudal rays (not segmented), segmented unbranched rays, and segmented branched caudal rays both dorsally and ventrally. Many vertebral and fin ray counts (except pectoral and pelvic rays) were taken from X-rays, and the last half-centrum bearing the urostyle is included in the vertebral counts. Both free neuromasts and presumptive canal neuromasts, where canals were not developed, were counted. Lines or groups of neuromasts reflect a history of annotation from symbols of Sanzo [33], Wongrat and Miller [34], and Anhelt et al. [15]. Our number (which corresponds with the illustration in Fig 4A) is followed here by a letter in parentheses corresponding to Anhelt et al. [15], NL indicates rows not shown by Anhelt et al.: 1 (s); 2(d); 3(c); 4(b); 5(ot); 6(z); 7(a); 8(x^1^); 9(x^2^); 10(as^1^); 11(os); 12(oi); 13(NL); 14(NL); 15(NL); 16(NL); 17(NL); 18(r); 19(u^1^); 20(tra); 21(u^2^); 22(q); 23(n); 24(la^2^); 25(la^2^); 26(o); 27(c^2^); 28(g). The two large organs (one above the other) along the upper edge of the preopercle were constant and not counted (i^3^).

We did not determine patterns of nervous innervation nor utilize the innervations-based terminology of Wongrat and Miller [34]. Extent of scale investment was assessed without counting the small, imbedded, and non-overlapping scales. Measurements were taken with dial calipers to the nearest 0.1 mm under a dissecting microscope and most fish were above 30 mm standard length (SL), with 15% being between 25 and 30 mm SL. Differences in pigmentary features could not be discerned in the fixed material examined and were not compared. Similarities as well as morphological and genetic differences between the species are discussed under *E*. *kristinae*.

### Nomenclatural Acts

Museum acronyms follow Sabaj Perez [35]. The electronic edition of this article conforms to the requirements of the amended International Code of Zoological Nomenclature, and hence the new names contained herein are available under that code from the electronic edition of this article. This published work and the nomenclatural acts it contains have been registered in ZooBank, the online registration system for the ICZN. The ZooBank LSIDs (Life Science Identifiers) can be resolved and the associated information viewed through any standard web browser by appending the LSID to the prefix "http://zoobank.org/". The LSID for this publication is: urn:lsid:zoobank.org:pub:F610F99F-347F-447E-A240-92AA29495215. The electronic edition of this work was published in a journal with an ISSN, and has been archived and is available from the following digital repositories: PubMed Central, LOCKSS.

### Material Examined

Localities utilized for meristics and morphometrics listed north to south along the coast including types; LACM numbers unless otherwise specified; M = male; F = female:

*E*. *newberryi*: Del Norte County: Smith River, 42683-2(15, counts only); Lake Earl, 42668–1, F: 10(31.8–37.9); Humboldt County; Humboldt Bay, 35334–1, M: 3(34.3–35.6); F: 2(34.9–36.5), 35335–5, M: 1(30.3); 42667–1, M: 1(33.1); F: 1(31.6); Mendocino County: Manchester State Beach/Brush Creek, 35322–1, M:2(28.6–35.3), 35134–4, M: 1(34.3); F: 3(36.4–38.5); 42666–1, M: 2(28.0–28.1); F: 1(28.2); Sonoma/Marin County line: Estero Americano, 37380–1, M: 4(29.6–35.8); F: 6(29.4–42.3); Santa Cruz County: Rodeo Gulch/Corcoran Lagoon, 42658–4, M: 5(30.9–33.8); F: 5(33.6–37.3); San Luis Obispo County: Arroyo de Corral, 42349–1, M: 5(31.7–35.8); F: 5(29.2–32.8); Morro Bay, 42348–2, M: 1(36.4); F: 3(29.4–36.1); 35573–1, M: 3(30.3–35.8); F: 5(30.4–34.3); Santa Barbara County: Santa Ynez River lagoon, 42343–1, M: 5(33.4–36.4); F: 5(35.5–42.3); Jalama Creek, 31425–2, M: 5(30.0–33.9); F: 5(31.0–37.2); Agua Caliente Canyon, 42340–1, M: 5(30.1–32.8); F: 5(29.7–38.2); Winchester/Bell Canyon, 42339–1, M: 2(33.6–35.1); F: 8(31.8–38.9); Ventura County: Ventura River, 35597–4, M: 2(34.4–37.0); F: 7(38.5–41.5); Los Angeles County: Malibu Creek, W55-273(15, counts only).

*E*. *kristinae*: Orange County: Aliso Creek, 42370–1 (20 counts only); San Diego County: San Mateo Creek, 57333–1, M: 1(28.3); F: 10(26.8–30.8)[paratypes]; San Onofre, 42692–2, M: 3(28.2–33.5); F: 4(32.7–37.5)[paratypes]; Las Flores/Las Pulgas, 57334–1, M: 3(23.8–26.2); F: 5(25.0–29.3)[paratypes]; 57334–2, F: 1 [Holotype] (29.3); one additional specimen, counts only [paratype]; San Luis Rey, 50478-1(15, counts only); Buena Vista, W53-235(15, counts only).

### Mapping

Verified records for both species are mapped based on museum specimens or other reliable documentation. See S1 Text for treatment of each locality mapped.

Four localities reported once but not documented by specimen records, or other information to provide definitive confirmation, are not plotted on the maps. They may be valid historical sites lacking Tidewater Gobies today: San Pedro, Los Angeles County reported by Eigenmann and Eigenmann in 1892 [36]; San Elijo Lagoon, San Diego County [37]; Carquinez Straits, upper San Francisco Bay [38], and Lake Merritt, Alameda County (visual records “…in the last few years…” by Jim Carlson as related verbally to Peter Moyle, in lit. 21 April 1975).

### Synonymies

Prior to observations of *Eucyclogobius* south of the Los Angeles Basin in 1939 and 1940 mention of Tidewater Gobies refer to the Northern Tidewater Goby *E*. *newberry* as delimited here. The synonymy in the main text includes, primary systematic treatments, misidentifications, and the synonymy of *E*. *kristinae* only. A more comprehensive compilation of synonymy and valid literature references for the genus and both species is appended (S2 Text).

### Analyses

Counts and measurements were analyzed with approaches mitigating the influence of size, sexual dimorphism [9], sampling limitations, as well as the local variation in morphology already known to exist [15] between the multiple lesser genetic subdivisions within *Eucyclogobius newberryi*. In the multivariate approach, discriminant function analysis (DFA), all measurements were globally transformed to eliminate the overall effect of size, and eliminate the specific reliance on a single measure to assess size. Separate DFAs were performed for species, sex, and species for each sex. Measured characters were then assessed individually without transformation using generalized linear models (GLMs) to understand the influence of effects of standard length, species, sex, and interactive terms for each character. This permitted identification of allometric relationships, not revealed in the discriminant approach and permitted identification of additional covariates excluded as redundant in species discrimination, but of interest in a more wholistic understanding of species difference.

Counts included fin spines and rays, vertebrae, and neuromast lines on the head and were treated independently from one another. Each was assessed for statistical difference between species using a non-parametric approach. Counted characters determined to be distinct at the 0.001≤p level between species was then further assessed to determine if all sampled localities were diagnosed effectively to species.

Finally, previous efforts to reconstruct the phylogeny and timing of divergence within East Pacific Bay Gobies [29,30] were analyzed to generate specific estimates of the divergence time of *Eucyclogobius newberryi* and *Eucyclogobius kristinae*.

#### Discriminant Analysis

To assess morphologic distinction between taxa, 45 measured characters from 142 individuals [115 *E*. *newberryi* (48 male, 69 female); 27 *E*. *kristinae* (7 male, 20 female)] were analyzed in a DFA. All characters in the matrix were first log_10_ transformed to standardize for variance, and then size corrected (S2 Table) using methods from Darroch and Mosimann [39], Jungers et al. [40] and Plogar et al. [41]. This consisted of calculating the arithmetic mean from all 45 morphological variables for each individual goby, then subtracting that value from each variable to create scale-free, or size corrected variables [41]. Mean value substitution, calculated from all individuals from the same geographical grouping (i.e. northern or southern species), was used in the few instances where data were missing from the matrix [42].

A linear DFA was implemented in SYSTAT 13.1 in order to determine which measured characters most effectively distinguished between these two species, and to test if these discriminating characters vary between sexes. Three analyses were conducted relative to species using the 45 morphological predictor variables and *E*. *newberryi* and *E*. *kristinae* as the categorical variable: 1) all individuals of both sexes pooled together, 2) females only, and 3) males only. A fourth analysis with sex as the categorical variable was also performed on pooled *E*. *newberryi* and *E*. *kristinae* samples. Prior probabilities were computed and assigned proportionally to the sample size of the group, due to unequal sample sizes for the two species. Stepwise selection was used to identify the subset of quantitative variables that most efficiently discriminates the two species among the designated samples. This method alternates between forward selection (based on F-to-enter criterion) and backwards elimination (based on F-to-remove criterion) by removing non-significant variables from the analysis before the next forward-selection step [43]. Stepwise discriminant analysis has been commonly used to analyze fish morphometrics and meristics [44–46] in addition to performing well with small sample sizes [43]

#### Generalized Linear Model Character Assessment

To provide a more comprehensive assessment of individual characters and compliment the DFA, GLM analysis was performed for each of the 45 measured characters except standard length. Standard length, species, sex, and the interaction terms species×length, sex×length, species×sex, and species×sex×length were included in the initial models as effects/predictors in the program JMP Version pro *12* (SAS Institute Inc). Each model was adjusted for false discovery rate. Subsequently, variables were removed in succession from the analysis and the model rerun proceeding from higher to lower order interactions following Winer et al. [47] until only effects that met a standard *alpha* (p<0.05) were retained. Exceptions were made in cases where an interactive effect included the variable that did not meet *alpha* (p<0.05). For example, sex would be retained if sex×length met the criterion and sex alone did not. Except for one case, 35 BETDFINS, residuals suggested normal distribution and the data were run without transformation (normal, identity) in JMP. Chi-squared values for effects in each model were tabulated by type of result found and categorized by standard *alpha* levels (p<0.001, 0.001≤p<0.01, 0.01≤p<0.05). It should be noted that the false discovery rate used in the analysis only pertains to multiplicity of tests within each model. In light of multiple tests, and the 45 models run, an *alpha* level of p<0.001 was employed for significance. Measured characters were plotted versus standard length (in JMP) such that the linear fits and confidence of the fit could to be compared for the two species and/or sexes.

#### Treatment of Counted Characters

Each counted character was examined using a bar chart and distinctions between species were assessed using a nonparametric test of central tendency (Wilcoxon Ranked Sums Test) appropriate for discrete data with potentially non-normal distributions. In light of multiple tests, an *a priori alpha* level of p<0.001 was employed. To determine whether meristic variables were diagnostic, or whether local variation interfered with diagnosis, a one-way ANOVA plot was generated for sampling sites for statistically significant counted characters. This was an expedient way of determining whether statistically significant results were consistently diagnostic for all samples. That is whether statistically significant results were also consistently diagnostic for all samples. No differences in meristic counts between sexes were observed.

#### Mitochondrial Sequence Divergence

Mitochondrial cytochrome b sequences (401-bp alignment) were used to compute minimum pairwise and mean between-group sequence divergences (p-distance) in MEGA v.6.06 [48], and to tally the number of fixed substitutions between *E*. *newberryi* and *E*. *kristinae*.

## Results

### Measured Characters

#### Discriminant Analysis

The discriminant analysis for species with sexes considered together properly classified 99% (115/116) of *Eucyclogobius newberryi* and 96% (26/27) of *E*. *kristinae* individuals using five characters (36 PECTLE, 16 POMXSPDO, 46 SFDIANO, 32 ANALLE, 42 BETPECO–see Fig 1 caption for character description) for a 99% classification rate of individuals to species. Three variables (38 SNMAXR, 18 SPDOANO, 9 ANALBL) were sufficient to classify 90% of individuals of both species to sex. Classification between species within sexes was also efficient. For females 96% (66/69) of *E*. *newberryi*, and 95% (19/20) of *E*. *kristinae* individuals were properly classified on the basis of 5 variables (24 SNOLPECO, 16 POMXSPDO, 32 ANALLE, 36 PECTLE, 47 MAXPECOL), and for males 98% (47/48) of *E*. *newberryi*, and 100% (7/7) of *E*. *kristinae* individuals were properly classified with 3 variables (36 PECTL, 26 ORBDIA, 38 SNMAXR) albeit with limited sample size.

#### Generalized Linear Models

The GLMs examined the suite of measured characters relative to predictors/effects: standard length, species, sex, and interaction terms species×length, sex×length, species×sex, and length×species×sex. Effects of models for species, species×length, sex and sex×length are reported in columns 1–4 respectively in Table 1. Results of GLMs for species and for sex are illustrated in Figs 1 and 2, respectively. Standard length was a critical term in the GLMs. However, it was not tabulated, as it was relatively and uniformly significant and was always included in final models where other effects met the *alpha* criterion for inclusion. In contrast, the interactive term length×species×sex did not meet the *alpha* level criterion for inclusion in any case and thus was not included in any final model and is also not reported in Table 1. The interactive term species×sex was significant in models for three characters. These results are not in the table, but are discussed further below.

**Table 1:** Results of Generalized Linear Models.

	1) Sp—*X*^*2*^	2) Sp*L—*X*^*2*^	3) Sex—*X*^*2*^	4) Sex*L—*X*^*2*^
**SPECIES EFFECTS**				
32 ANALLE	30.33***	7.93**	--	--
16 POMXSPDO	27.06***	9.10**	10.24**	--
42 BETPECO	20.32***	6.31**	--	--
19 PELOSFDO	22.14***	3.90**	10.10**	6.07*
3 OCCSP	14.86***	11.78***	--	--
17 SPDOPEO	12.60***	4.30*	--	--
36 PECTLE	12.15***	5.07**	#	--
25 SNORB	#	37.41***	6.78*	--
30 SAFTDRA	10.56*	17.16***	9.14**	--
40 LMAXRPE	11.40**	4.87*	--	--
41 RMAXLPE	11.52**	5.25*	#	4.39*
33 CAUDLE	10.98**	--	4.39*	--
5 SFTDB	10.22**	--	--	--
47 MAXPECOL	6.19*	--	--	--
23 ANIDC	5.06*	--	--	--
26 ORBDIA	4.51*	--	--	--
**SPECIES and SEX EFFECTS (similar in** *X*^*2*^**)**				
9 ANALBL	16.27***	--	25.17***	--
46 SFDIANO	19.13***	--	17.65***	--
15 OCCPELO	#	4.98*	8.96**	--
21 SFTOANI	6.70*	--	4.79*	--
**PREDOMINANTLY SEX EFFECTS with Allometry**				
38 SNMAXR	#	5.41*	142.13***	32.63***
37 SNMAXL	5.12*	--	137.68***	29.08***
39 MOUWIDE	21.07***	8.64**	60.02***	37.21***
27 POSTORB	--	--	74.42***	13.92***
28 POSTOPER	--	--	51.59***	12.31***
24 SNOLPECO	7.24**	4.33*	64.52***	7.20**
7 SNOPELO	--	--	28.01***	5.66*
11 OCCMAX	#	4.87*	5.85*	11.74**
**PREDOMINANTLY SEX EFFECTS with minimal Allometry**				
44 RPECLCAU	4.83**	6.56*	28.40***	6.37*
43 LPECLCAU	10.63**	7.24**	23.68***	9.81**
10 ANFILCO	--	--	22.77***	--
8 PELOANO	3.90*	4.20*	17.68***	--
18 SPDOANO	--	--	12.31***	--
35 BETDFINS^1^	--	--	26.57***	--
6 SFTDUCB	--	--	11.35***	--
2 SNOCC	--	--	9.48**	--
22 SFTDIVC	--	--	7.46**	5.55*
14 DEPCAO	--	--	6.53*	--
45 RMAXRPEC	--	--	4.43*	--

Chi-square statistic for effects in the model are tabulated. Standard *alpha* levels are shown by asterisks (*** p≤0.001, ** 0.001<p≤0.01, * 0.01<p≤0.05 and -- 0.05<p-removed from model). When the measure is larger in *E*. *newberryi* or males, the effect reported is in bold type; when measures are larger in *E*. *kristinae* or females, the effects are underlined. Column 1, effect of species; column 2, effect of species×length interaction (allometric difference between species); Column 3, effect of sex; Column 4, effect of sex×length interaction (allometric difference between sexes). The interaction of sex and species (differences in dimorphism between species) is reported in the text. The effects of length are not shown as there is always a strong relationship between the measured standard length and the other measured characters. The measures are grouped by the most dominant effects as determined by *X*^*2*^ of the effect (*n* is nearly identical between models). Species, species×sex, sex, and sex×length (allometry) and sex alone were the emergent sets of explanatory effects. ^1^Character 35, BETDFINS, was not normally distributed due to a number of zero values where dorsal fins were continuous, zeroes were removed prior to analysis.

Species had an effect at p<0.05 on 23 measured characters, and 10 of these were at the p<0.001 level (Table 1). This included the 5 characters that best differentiate species in the discriminate analysis (Fig 1). As is to be expected, additional characters that incorporated redundant discriminatory information that were excluded in the discriminant analysis are revealed as significant here. These include characters that reinforce an interpretation of greater height and girth in *E*. *kristinae*, and longer fins in *E*. *newberryi* (Fig 1). Species×length had effects on 19 measured characters (3 of these at the p<0.001 level). These species×length differences in the model can be interpreted as differences in allometry between species. The greater increase with size in *E*. *kristinae* of character 30, the initial ray of the 2^nd^ dorsal fin, may be the most noteworthy of these allometric characters.

Sex had an effect at p<0.05 for 28 characters, 16 of these at the p<0.001 level. Sex×length was an effect for 13 characters, 5 of these at the p<0.001 level. It is noteworthy that of the characters that are distinct between sexes, a suite of jaw and head characters are larger in the male, and grow allometrically (Table 1). These are presumably involved in the burrowing behavior of the adult males. Sexually distinctive characters that don't appear as allometric include the gap between the two dorsal fins, and the space between the caudal fin and the dorsal and anal fins. Three characters not shown in Table 1 reveal effects of species×sex interactions (30 SAFTDRA 7.82**, 41 RMAXLPE 4.27*, 39 MOUWIDE 11.3***- notation of *X*^*2*^ and asterisks for *alpha level* and underlining as in Table 1). Thus *E*. *kristinae* shows larger dimorphism in mouth width (Fig 2D) with substantially greater mouth-width in males. Otherwise, *E*. *kristinae* and *E*. *newberryi* show limited differences in sexual dimorphism between species. The low sample size of *E*. *kristinae* males in the analysis may limit the statistical power to identify this class of interaction.

### Counted Characters

#### Mitochondrial Sequence Divergence

In addition to tree reconstruction based on D-loop and cytochrome b sequences Dawson at al. [10] estimated divergence times based on cytochrome b. As it was not reported previously, we calculated minimum pairwise sequence divergence (p-distance) between *E*. *newberryi* and *E*. *kristinae* in a 401-bp fragment of mitochondrial cytochrome b to be 4.1% [48]. Mean divergence between the two groups was 4.7%. Assuming a 0.5–1% mitochondrial divergence per million years, a conservative estimate for vertebrates, Dawson et al. [10] inferred the two lineages diverged ~2–4 million years ago (Mya), numbers consistent with our sequence divergence calculations. The 401-bp fragment also contains 11 fixed substitutions between the two lineages. A DNA barcode based on these differences may be used to distinguish the two species as follows, where the first nucleotide is *E*. *newberryi* and the number is the nucleotide position relative to the complete mtDNA genome of *E*. *newberryi* (Accession no. KP013101.1); C:T– 14443, C:A– 14479, G:A– 14575, C:T– 14644, G:A– 14686, C:T– 14689, G:A– 14702, A:G– 14725, T:C– 14759, G:A– 14785, T:C– 14806.

## Taxonomy

### Synonymy

#### Genus Eucyclogobius Gill, 1862

*Eucyclogobius* Gill, 1862:[49]: 279, 330, 332, brief original description, type species *Gobius newberrii* Girard 1856:[1]: 136, by original designation and also monotypic; Gill, 1863:[50]: 264, 265, list; Bleeker 1874:[51]: 298, 319, classification, type *Gobius newberrii*; Jordan and Gilbert, 1882:[52]: 331, squamation compared to *Microgobius emblematicus*; Jordan and Gilbert 1883:[53]: 637–638, subgenus of *Lepidogobius;* Jordan, 1887:[54]: 894, list, subgenus of *Lepidogobius*; Jordan and Eigenmann 1887:[55]: 517, subgenus of *Lepidogobius*; Eigenmann and Eigenmann 1888:[56]: 69, listed, subgenus of *Lepidogobius*; Jordan and Evermann 1896:[57]: 459, list; Jordan and Evermann 1898:[58]: 2248, description, comparison with *Lepiogobius*; Jordan 1923:[59]: 225, classification; Hubbs 1926:[60]: 3, characteristics, distinct from *Lepidogobius*; Barlow 1961:[61]: 424, related to other northeastern Pacific estuarine gobies; Norman 1966:[62]: 405, 415, key to world goby genera, characters, list; Birdsong et al. 1988:[22]: 187, axial skeleton, gobiid classification; Eschmeyer and Bailey 1990:[63]: 144–145, catalogue of genera of recent fishes; Eschmeyer 1990:[64]: 488, list in classification; Burr and Mayden 1992:[65]: 23, list of North American freshwater genera; Pezold 1993:[66]: 640, classified in subfamily Gobionellinae of family Gobiidae; Nelson 1994:[67]: 416, in subfamily Gobionellinae of Gobiidae; Larson 2001:[68], phylogenetic relationships; Thacker 2003:[69]: 357, 359, 360–362, molecular phylogeny; Nelson et al. 2004:[70]: 171, list; Nelson 2006:[71]: 423, in subfamily Gobionellinae of Gobiidae; Kindermann et al. 2007:[72]: 13–56, osteology; Thacker 2009:[23]: 96, molecular phylogeny, in family Gobionellidae; Ruber and Agorreta 2011:[73]: 31, 40–41, microsatellite studies, gobiid molecular phylogenies; Pezold 2011:[25]: 94, list, North Pacific gobionellid genera; Van Tassell 2011:[26] 143, list, Gobiiformes of the Americas; Thacker 2013:[24]: 371, member of North Pacific group; Tornabene et al. 2013:[27]: 4, 11, relationships; Agoretta et al. 2013:[28]: 627, phylogenetic relationships; Ellingson et al. 2014:[29]: 465, 467, 472–475; phylogeny, morphological convergence with western Pacific species.

#### Diagnosis

The genus *Eucyclogobius* is diagnosed morphologically from other genera in the eastern Pacific Lepidogobius clade (*Lepidogobius*, *Ilypnus*, *Quietula*, *Clevelandia*, and *Evermannia*; see Ellingson et al. [29] by: adult supraorbital canals separated across dorsal midline vs. connected with or without a pore; gill opening ends below upper pectoral fin origin; dorsal fins closely approximated or confluent vs. distinctly separated; second dorsal and anal fin elements fewer, usually 10–12 vs. 13 or more; maxillaries not prolonged posteriorly vs. prolonged; cleithral shoulder papillae low and rounded vs. elongate; squamation lacking on belly, nape, isthmus and head vs. present in one or more of these areas in addition to the body; scales cycloid and largely non-overlapping vs. scales overlapping on body; specifically adapted to brackish lagoonal or microtidal habitats vs. more saline and tidal estuarine and marine environments; female dominant reproductive behavior vs. male dominant in other eastern Pacific species known [9,16].

### Eucyclogobius newberryi (Girard, 1856)

#### Northern Tidewater Goby

*Synonymy and Valid Literature References*: *Gobius newberryi* Girard, 1856:[1]: 136, original description as “…a brief account, extracted from final reports…”; Girard 1857:[2]: 539–541, figures 5–8, plate 25, misspelled as “*newberrii*,*”* description, illustration, specimens from Tomales Bay; Girard 1858:[3], 1859:[4]: 128, [repeated] descriptions in editions of the western railroad surveys, types USNM 360, 24 specimens from Tomales Bay; Gunther 1861:[74]:77, list, description from Girard, as “*newberrii*”; Steindachner 1879:[75]: 135–137, description of specimens from artesian springs, Santa Monica, California, as “*newberrii*”.

*Lepidogobius newberryi*, Gill 1859:[76]: 14, list; Lockington 1880:[77]: 18, list of California fishes; Jordan and Gilbert 1881:[78]: 455, list, table of Pacific coast fishes; Jordan and Gilbert 1883:[53]: 637–638, description, range, comparisons; Jordan 1887:[79]: 894, list, included within subdivision or subgenus *Eucyclogobius*; Jordan and Eigenmann 1887:[55]: 502–503, 515, 517, keys, description, list; Eigenmann and Eigenmann 1888:[56]: 69, list, as “*newberrii*”; Eigenmann and Eigenmann 1892:[36]: California list, footnote record for San Pedro, no extant specimens known; Ginsburg 1945:[80]: 134–137, 139, caudal fin ray counts.

*Eucyclogobius newberryi*, Gill 1863:[50]: 264–265, synonymy; Bleeker 1874:[51]: 298, 319, classification; Jordan and Gilbert 1881:[78]: 53, listed; Jordan and Gilbert 1882:[52]: 331, squamation compared to *Gobius* (now *Microgobius*) *emblematicus*; Jordan and Evermann 1898:[58]: 2248, description, range, confined to freshwater; Evermann and Clark 1931:[81]: 58, 63, California habitat, list; Shapovalov and Dill 1950:[82]: 387, California list; Shapovalov et al. 1959:[83]: 174, California list; Kimsey and Fisk 1960:[84]: 474, key California freshwater fishes; Norman 1966:[62]: 405, 415, type species of genus; Miller and Lea 1972:[85]: 186–187, key, characters, range; Miller and Lea 1976:[86]: 240, range extended; Moyle 1976:[87]: 85, 344–346, key, biology, distribution; Hubbs et al. 1979:[88]: 44, California list; Eschmeyer et. al. 1983:[89]: 262, pl. 19, illustration, characters, range; Swenson 1999:[9]: biology, conservation, behavior, distribution; Anhelt et al. 2004:[15]: 385, 398, variation in head canal development; Kindermann, et al. 2007:[72]: 13–56, osteology, possible relationships; Earl et al. 2010:[12]: 103–114, phylogeography, distinctness of southern population.

*Gillichthys mirabilis*, Starks and Morris 1907:[90]: misidentification, Mendocino County record described as “Specimens examined from Mendocino County, 1¾” in length, contained mature eggs.”; Snyder 1938:[91]: 358, misidentification, Waddell Creek lagoon.

*Eucyclogobius* (N), Ellingson et al. 2014:[29]: 472, morphological convergence with western Pacific species of *Gymnogobius*.

*Type Specimens of* Eucyclogobius newberryi: Girard’s original description of the species was abbreviated [1], and subsequent descriptions in 1857 [2] and in two editions of the Pacific Railroad Surveys [3,4] were more detailed and specified USNM 360, 24 specimens as the types of his *Gobius newberryi* collected by E. Samuels in Tomales Bay, or adjacent coast. Girard described several other California coastal marine fishes taken by Samuels in Tomales Bay and in or near Petaluma, northern San Francisco Bay (also called San Pablo Bay). By July of 1984 USNM 360 consisted of three gauze-wrapped groups of fish with the data: “California, Petaluma. Samuels” (Richard Vari, Smithsonian Institution, letter July 10, 1984). In 1991 Helen Larson found these three wrapped lots of 20, 20, and 23 specimens, respectively (personal communication, 2013), to all be *Eucyclogobius newberryi*. These lots are accompanied by small numbered paper labels that are now disassociated from individual specimens. Without evidence to the contrary (Jeffrey Williams, USNM, personal communication), the lot of 23 are presumed to be the types mentioned by Girard and the missing specimen may be the one illustrated by Girard [2] and not reunited with the rest of the type lot. The other two lots were possibly added as part of the 185 caudal ray counts taken later by Ginsburg [80] who then worked at the Smithsonian.

The 23 type specimens have faded to a uniform medium tan color and are very flattened from being wrapped tightly with impressions of cheesecloth on their sides and pectoral fins adherent to the body. All have two distinguishing characteristics of *Eucyclogobius*, namely a distinct depigmented distal one-quarter to one-third of the spinous dorsal fin and the upper limit of the gill opening ending below the upper edge of the pectoral fin base. The USNM catalogue number 360 is retained for the lectotype selected, a 39.2 mm SL female, with measurements and counts as follows: head length (L) 10.4; predorsal L 14.8; prepelvic L 10.7; preanal L 25.6; body depth at second dorsal origin 6.8; caudal peduncle depth 4.3; head depth 6.3; snout L 2.3; horizontal eye diameter 2.3; Posterior margin of eye to posterior end of opercle 6.1; Posterior margin of eye to posterior edge of preopercle 3.5; interorbital width 1.6; mouth width 3.9; Snout to posterior end left maxillary 4.9; first dorsal base 7.4; space between dorsal fins 0.2; second dorsal base 9.8; anal base 10.7; Right pectoral fin L 7.0 (left disfigured from wrapping); pelvic L 7.5; caudal fin L 8.4. Dorsal spines 7, second dorsal fin and anal fin elements each 11, precaudal 16 and caudal 18 for a total of 34 vertebrae. Maxillary extends back to below the eye but not beyond a vertical line through the posterior margin of the orbit. The dorsal and anal fins are darkly pigmented with melanophores and the head and body have scattered melanophores. Four small saddle-like groups of melanophores lie along the dorsal midline from the back of the head to the first dorsal fin. The upper 6–7 pectoral rays are pigmented and the caudal, left pectoral, and pelvic appear unpigmented. The slender conical teeth are in two to three rows anteriorly with the outer slightly larger. The remaining 22 specimens are designated paralectotypes and catalogued as USNM 408102, 23.6 to 39.7 mm SL. They are all very pale brown with darkened dorsal and anal fins. The top of the skull of two specimens has been dissected open. Counts from X-rays (excluding pectoral rays) from all 23 fish are summarized in S1 Table.

### *Eucyclogobius kristinae* new species, Swift, Spies, Ellingson and Jacobs

#### Southern Tidewater Goby

*Eucyclogobius kristinae*, Swift, Spies, Ellingson, and Jacobs, new species, urn:lsid:zoobank.org:act:CC641CE5-7DAE-466E-BA3F-D23994068F2B.

*Gillichthys mirabilis*, Metz 1912:[92]: 41, misidentification, record from Aliso Creek, Laguna Beach, Orange County.

*Eucyclogobius newberryi*, Miller 1939:[5], 1943:[6], records from San Juan Creek, Orange County; Swift et al. 1989:[16]: 1–19, in part, biology, distribution, illustration; Earl et al. 2010:[12]: 103–114, phylogeography, distinctness of southern population; Ruber and Agoretta 2011:[73]: 31–41, in part, reanalysis of gobiid molecular phylogeny; Van Tassell 2011:[26]: 143, in part, list of Gobiiformes of the Americas.

*Eucyclogobius* (S), Ellingson et al. 2014:[29]: 472, convergence with western Pacific species.

#### Holotype

**Fig 5:**
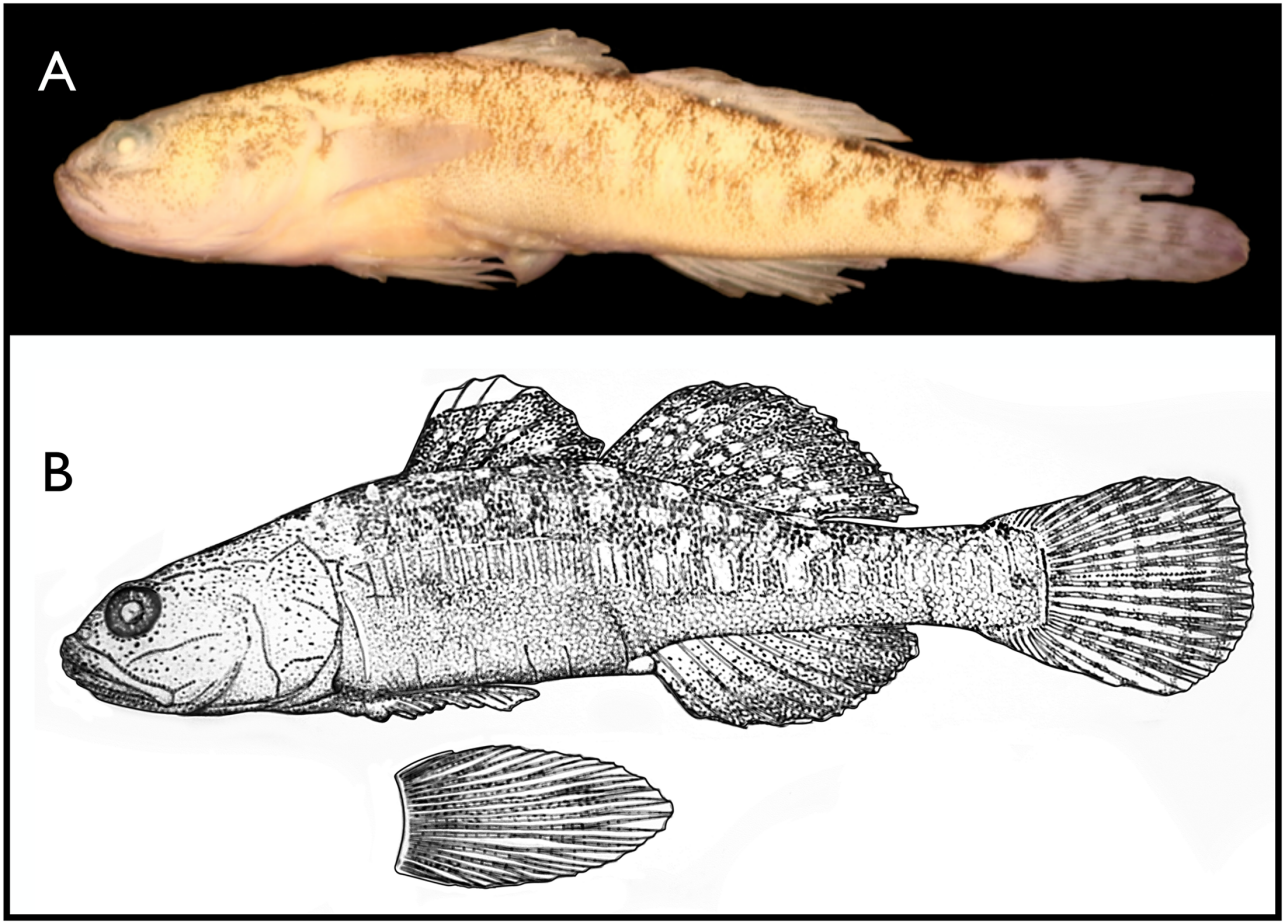
Panel A, Photograph of holotype of *Eucyclogobius kristinae*, n. sp., LACM 57334–2, 29.3 mm Standard Length from Las Flores Canyon lagoon on Marine Corps Base Camp Pendleton on 29 July 2011. See text for other data. Panel B, Illustration of pigment patterns and some neuromast lines on the head and body of *Eucyclogobius kristinae n*. *sp*., LACM 42639–1, 33 mm SL, Aliso Creek, Orange County, CA [16].

LACM 57334–2, female, 29.3 mm SL (Fig 5), California, San Diego County, coastal lagoon at mouth of Las Flores Canyon (Las Pulgas Canyon on some maps), about 15 km northwest of Oceanside, centered on 32° 17’ 25.29” N; 117° 27’ 51.40” W, 29 July 2011, by Kevin Lafferty, Mike Rouse, Cary Galst Cavalcante, and Brenton Spies. Dorsal fin VI, 12; Anal 10, Pectoral 19, 19; Pelvic I, 10, I; Caudal rays 10 + 2 + 6 + 5 + 2 + 9 (see methods), total branched 11, total caudal rays 34; vertebrae 16 + 18 = 34; gill rakers 3 + 6, 9 total; anterior exposed left supraorbital neuromasts 11. Fixed originally and maintained in ethyl alcohol.

*Paratypes*: LACM 57334–1, 8 (23.8–28.3), data as holotype; LACM 57333–1, 11 (26.4–30.8), CA, San Diego County, San Mateo Creek lagoon, just south of Orange County line, 29 July 2011, date and collectors as holotype; LACM 42692–2, 7 (28.2–37.5), CA, San Diego County, San Onofre Creek lagoon, about 2 km south of Orange County line, 3 December 1981, Camm C. Swift. No other types are designated but LACM, SIO, CAS, and UMMZ, have several series of non-types from earlier samples from these southern populations that have been examined.

*Diagnosis*: *Eucyclogobius kristinae* is distinguished morphologically from the only other species in the genus, *E*. *newberryi*, by reduction (in fish over about 25 mm SL) of the anterior supraorbital canal and concomitant increase in number of exposed neuromasts (see analyses above), 8–12 in adult *E*. *kristinae* vs. 5–8 in *E*. *newberryi* (Fig 4B). The Southern Tidewater Goby averages about one fewer pectoral fin ray (18–19 vs. 19–21) and branched caudal rays 10–11 (9–12) vs. 12–13 (11–14) due to reduction in this count on the lower half of the caudal fin (Fig 3B). Morphologic measures proved efficiently diagnostic in combination (Fig 1B–1F). These include a longer anal spine and measures that contribute to greater girth, more anterior placement of pelvic fins and a more upturned mouth in *E*. *kristinae*, while *E*. *newberryi* has a more elongate snout with a more terminal mouth, longer pectoral fins and somewhat more elongate dorsal, anal, and caudal fins. Numerous fixed molecular characters have been identified between the two species, both in mitochondrial sequence [10], as well as in dramatic length difference and amplification of microsatellite loci [12]. Thus, there are many means of efficient molecular diagnosis via PCR amplification assay.

#### Description

Measurements for holotype and paratypes of *E*. *kristinae* on (S1 Table). Body elongate with width and depth greatest about one-third of the standard length back from tip of snout; body rounded, only slightly deeper than wide, more compressed after preservation; up to 45 mm SL or 55 TL; head 25–30% of SL, snout and eye subequal; interorbital width less than eye diameter. Mouth slightly upturned, maxillary extending back to vertical through middle of eye, not prolonged but mouth larger in males (Fig 2). First and second dorsal and anal fins subequal in height, caudal fin length about 18% of standard length. First and second dorsal fins narrowly separate to slightly confluent, when separate space less than 20% of length of first dorsal base. In 13 of 30 specimens (31–36.6 mm SL) from the San Luis Rey River lagoon from 1958 (LACM 50478–1 [ex. UCLA W58-1]) the last fifth or so of the first dorsal fin base and insertion was deflected to the left (5) or the right (8) of the midline, a condition not observed otherwise among hundreds of specimens of both species. Pelvic fin an elongated oval extending posteriorly about half the distance to the anal origin, anterior margin of disc a thin translucent membrane with smooth, continuous slightly concave edge. Anterolateral edge of lower limb of cleithrum with 0–4 low rounded fleshy papillae. Nasal capsule slightly bulbous and each nostril slightly raised on cone shaped elevations, occasionally slightly tubular. Upper end of gill opening ends just below the upper edge of pectoral fin, lower gill cover broadly attached to isthmus, slightly in front of lower pectoral base.

Upper and lower jaw teeth with outer row of slender, slightly recurved conical teeth followed by two to four inner rows of smaller teeth near symphysis. Inner tooth rows diminish to one row laterally. No vomerine, palatine, or pteryoid teeth. Upper and lower lips with infolded groove along the inner edge of the lips extending for 70–80% of the mouth margin. Gill rakers low and rounded. Scales thin and cycloid, mostly non-overlapping and on sides of body and caudal peduncle in adults, usually absent mid-dorsally, mid-ventrally near median fins and absent from belly, isthmus, nape, and head. Scales lacking until about 25 mm SL.

Meristics for both species are summarized in Supplementary Information (S3 and S4 Tables). First dorsal spines 4–8 (usually 6 or 7); second dorsal 9–13 elements usually 10–11 in *E*. *kristinae*, 11–12 in *E*. *newberryi*; anal fin 9–12 (10–11); pectoral fin rays 17–21, usually 18–19 in *E*. *kristinae*, 19–-21 in *E*. *newberryi*; pelvic elements 12 including a small slender spine on the anterior edge of each side; one specimen had one less branched ray; branched caudal rays 9–14, usually 11–12 in *E*. *kristinae*, 12–13 in *E*. *newberryi*; total caudal rays (procurrent plus unbranched plus branched) 30–39 (usually 33–36). A few total caudal ray counts below 30 are likely anomalous. Vertebrae 32–36, usually 34–35; precaudal or abdominal 15–16; caudal 17–19 (usually 19). Branchiostegal rays 5; gill rakers 9–11 in *E*. *kristinae*, 8–12 in *E*. *newberryi* and usually a count of 9 or 10 in both species, 3 upper and 6 or 7 lower limb rakers.

Only the posterior segment of each supraorbital canal develops in this species [15] and then only in adults. The two supraorbital canals also do not meet in the dorsal midline between the eyes. Adult *E*. *newberryi* usually have both supratemporal canals developed anteriorly and can have an interorbital connection or commissure with one pore in the middle if joined but usually with two pores opposite each other without a complete commissure. Fish less than about 25 mm SL usually lack canal development. No other lateral line canals developed.

Free neuromast organs largely in longitudinal rows on the head (Fig 4); on the body (Fig 5B) in a series of vertical rows, one per myotome, down the mid-sides of the body, a few short rows extending up the lower abdomen between the pelvic origin and cloaca, and in two lines extending on to membranes of the upper half of the caudal fin, separated by two branched rays. A few vertical rows also occur on the operculum and head above the operculum. The largest of these organs is about three times the diameter and size of the smallest. The largest on the inner row of the lower jaw and preopercle, under the eye, and on the snout presumably represent or are homologues of the lateral line organs enclosed in canals in other gobies. The variation in number of these organs is shown for some rows that could be reliably tabulated (S4 Table). One pair of larger organs along the upper edge of the preopercle and another pair between the anterior ends of the mandibles and just behind the symphysis are illustrated (Fig 4) but not tabulated since they were invariant. A pair of larger organs on the upper opercle were inconsistently present (No. 17). Neuromast lines were similar to those illustrated by Ahnelt et al. [15] and they show one set not counted here, a paired longitudinal series in the predorsal area.

Pigmentation in preserved material of larval and early juvenile fish is very similar in both species and was described in Swift et al. [16] and Watson [93] from material of *E*. *kristinae* and by Spies et al. [94] for *E*. *newberryi* from San Gregorio Creek lagoon, San Mateo County. Nine to eleven large stellate melanophores uniformly spaced mid-dorsally from nape to caudal base in late larvae and small juveniles form 7–9 dorsal saddles or blotches in larger juveniles and adults. Adult preserved specimens with pale white or slightly grayish or yellowish ground color with a wash of very fine punctuate melanophores (individual cells concentrated). The clusters or concentrations of expanded melanophores mid-dorsally usually lie: 1) between occiput and first dorsal fin, 2) at origin of first dorsal fin, 3) under the middle of the base of the first dorsal, 4) between the dorsal fin bases, 5) two equally spaced along second (soft) dorsal base, 6) one at the second dorsal insertion, 7) one between second dorsal insertion and dorsal caudal origin, and 8) one at caudal origin. The saddles under the dorsal fins are larger and better defined than those posterior and anterior. Five to ten pairs of diffuse blotches evenly distributed mid-laterally on the body; a third horizontal row of indistinct blotches lies between the mid-dorsal saddles and the mid-lateral blotches. In larger fish, the three longitudinal rows of blotches on the body (mid-dorsal, mid-lateral, and dorsolateral) loosely to densely coalesce leaving one or two irregular longitudinal rows of intervening clear or whitish areas appearing as spots sometimes elongated into vertically oriented pale vermiculations. A ventral saddle, posterior to the insertion of the anal fin, overlies the narrow midventral line of melanophores from anal origin to caudal base. This ventral line occasionally has one or two clear unpigmented breaks in the adult. The ventral procurrent caudal rays largely unpigmented with a patch of melanophores just above them. Caudal base with two additional concentrations of melanophores oriented vertically and just under the dorsal procurrent rays, the latter largely unpigmented also. Unpigmented spaces or windows lie in front of the three caudal blotches as well as behind them over the proximal ends of the caudal rays.

The caudal fin with five to six dark, equally spaced vertical bars about as wide as their interspaces, consisting of patches of melanophores mostly on the fin rays. The rays on the upper half or so of the pectoral fin lined with melanophores and a patch of melanophores lies on the dorsal one-third to one- half of the outer fleshy pectoral fin base. The second dorsal and anal fins are dusky with a more or less uniform covering of melanophores but with a narrow unpigmented distal edge (Fig 5). The second dorsal with one or two longitudinal and parallel rows of clear or pale spots, one per inter-radial membrane beginning in the middle of the anterior edge, descending to near its base posteriorly. The first dorsal is usually darker, has a wider distal unpigmented area equaling one quarter to one third the height of the fin, and sometimes with one or rarely two horizontal rows of clear spots also descending posteriorly. The marginal one to three rays of the pelvic fin can have some melanophores.

Head uniformly covered with dark melanophores, area over the brain slightly darker; cheeks and upper one third or so of opercle also dark along with margins of upper and lower jaws and anterior one-third or so of the ventral surface of the lower jaw. Vascularization (veins and/or arteries) on the roof of the mouth darkly pigmented and the testes enclosed in layer of dark melanophores.

In life males and females are similar in color and pattern as described above, but the background color is translucent grey, tan, or olive. The pale spots and vermiculations on the body and narrow unpigmented areas (lacking melanophores) on the median fins are white, cream, or pale orange. Sometimes they appear bluish or iridescent, probably due to the reflection of the sky commonly occurring during field examination of live fishes [95]. In breeding individuals these colors are intensified in the males and females. In addition, in courting females the dorsal and anal fins and the body between them can become solid black with the fins retaining the pale or colored edges. Males do not develop the very black condition as far as is known, and both sexes often show a whitish, cream, or light orange pigmentation on the marginal pelvic fin rays.

On 24 June 1989 a large adult individual *E*. *newberryi* taken and released at the mouth of the Santa Maria River that was partly xanthochroic. Most of the head, body and first dorsal fin were bright yellow. The very top of the head and eye was black. The anterior two thirds of the second dorsal was yellow with the posterior one-third black. Most of the anal fin was black except for a yellow anterior edge. The caudal peduncle was black along with the body between the posterior quarter of the second dorsal and anal fin bases. The caudal fin was yellowish-orange with a narrow black dorsal edge.

#### Distribution

**Fig 6:**
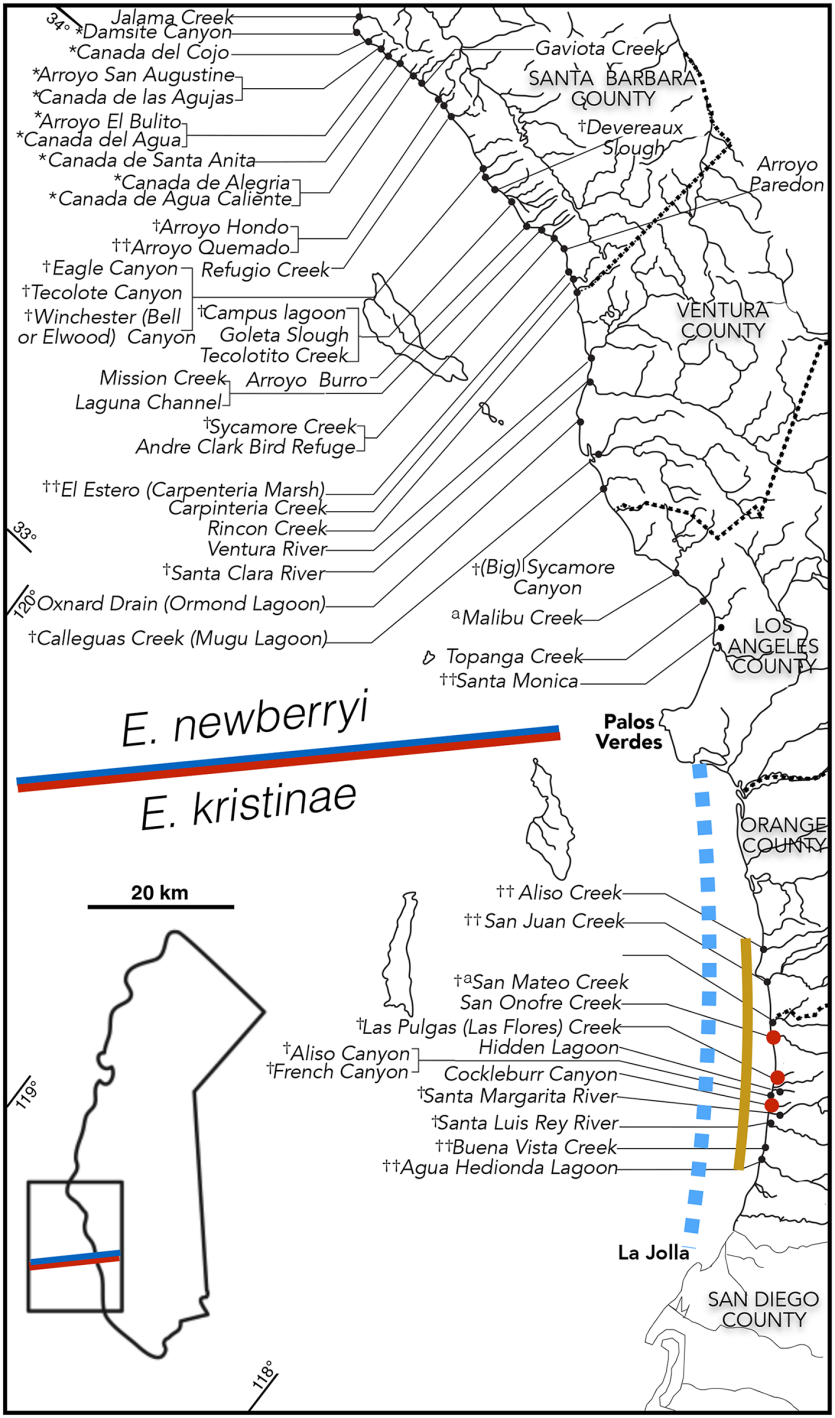
Map of the distribution of the Northern and Southern Tidewater Gobies across southern California. Northern Tidewater Goby in southern Santa Barbara, Ventura, and northern Los Angeles counties. Southern Tidewater Goby in Orange and San Diego counties. Blue/red line shows the geomorphic southern range limit of *E*. *newberryi* and the northern limit of *E*. *kristinae*. The high headlands at La Jolla and Palos Verdes appear to provide a geomorphic limit on the natural range of *E*. *kristinae*. The tan line demarcates the historic range of *E*. *kristinae* from Aliso Creek (Orange County) to Agua Hedionda (San Diego County). Red dots indicate the three localities where *E*. *kristinae* was recovered in recent surveys, highlighting the precarious status of this species. *Not sampled last two years. †Not recovered in recent sampling. ††Habitats that appear no longer viable due to anthropogenic modification. ^a^Sites of attempted reintroduction. Maps covering *E*. *newberryi* distribution in northern and central California are presented in supporting information (S1–S3 Figs).

Until the mid-20th century the Tidewater Goby was recognized from Santa Monica, Los Angeles County northward to the northwest corner of California. In 1939 and 1940, fish taken south of the Los Angeles area were recognized as *Eucyclogobius newberryi* by Miller [5,6] and new collections expanded the range south into San Diego County by the 1950s (UMMZ, UCLA, SIO, CAS). These southern fish are recognized here as *E*. *kristinae*. Metz’s 1915 record [92] of the Longjaw Mudsucker, *Gillichthys mirabilis*, from Aliso Creek, Orange County, was based on misidentified *E*. *kristinae*. A 1950 study purportedly of *Eucyclogobius* biology in Peñasquitos Lagoon, San Diego County [96] was based on the Longjaw Mudsucker [16]. Rechnitzer [37] referred to Tidewater Gobies at San Elijo Lagoon, but no confirmatory samples are known. In 1892 Eigenmann and Eigenmann [36] listed a San Pedro locality (as *Lepidogobius newberryi*) but specimens to support this record have not been found. Thus, verified records of *Eucyclogobius newberryi* extend from the mouth of the Smith River, Del Norte County, south to Santa Monica, Los Angeles County (Fig 6; S1 Text; S1–S3 Figs) and those for *E*. *kristinae* extend from Aliso Creek, Orange County south to Agua Hedionda Lagoon, San Diego County (Fig 6). Data are current as of October 1, 2015.

Over the last 30 years, *Eucyclogobius kristinae* has been restricted to 9 localities in northern San Diego County from San Mateo Creek near the Orange County line southward to the mouth of the San Luis Rey River. All but the San Luis Rey River lie on Marine Corps Base Camp Pendleton. Several of these localities have histories of local extirpations and, as of December 21, 2015, sampling in the last 3 years has documented the presence of the species in only three small sites limited to Camp Pendleton (Fig 6).

Many localities for both species have been extirpated with some naturally recolonized, and artificial reintroductions have been attempted in a few instances. Most of the losses have been in southern California and the San Francisco Bay area [13]. Several natural gaps also occur in stretches of coastline with steep shores where coastal lagoons do not develop, such as the Big Sur area from the Salinas River, Monterey County south to and including Arroyo de la Cruz in northern San Luis Obispo County. The smaller streams in this area are often too steep and lack fine substrates to develop barrier sand berms and stable coastal lagoons. The Northern Tidewater Goby also has never been recorded from several larger streams north of San Francisco Bay, the Russian, Gualala, Garcia, Navarro, Albion, Big, Noyo, and Mattole rivers, nor in Oregon just a few miles north of the northernmost locality in the Smith River. Until recently this list of absences included the Mad and Klamath rivers (S1 Text; S1 Fig) where the species has been recorded in the last year. It is not known if these are permanent populations or dispersing individuals. Occurrences throughout the range of both species often correspond to coastal littoral cells of sediment movement as described by Habel and Armstrong [97], where sediment is sufficient for berm formation and lagoon closure at the mouths of streams.

#### Etymology

The species name is feminine in honor of Kristina D. Y. Louie whose untimely death in 2004 cut short a promising career dedicated to conservation genetics. Her Ph.D. dissertation and associated work contributed greatly to our studies of eastern Pacific phylogeography [11, 12, 98, 99], as well as to a novel re-interpretation of the placement of Wallace’s Line across the islands of Indonesia [100].

## Discussion

### Phylogenetic Placement, Distinction, and Antiquity

#### Phylogeny of the Group

The two species of Tidewater Goby are part of a larger group of temperate North Pacific Bay Goby genera disjunctly distributed across the North Pacific [29]. One genus, *Evermannia*, ranges southward into the tropics in the eastern Pacific [101] and no western Pacific species does so [102]. Birdsong et al. [22] included many of these genera in their informal “Chasmichthys” and “Astrabe” groups based on the posterior placement and arrangement of the dorsal fin pterygiophores. Additional morphological synapomorphies for this North Pacific, largely temperate group of gobies including the northeastern Pacific genera *Eucyclogobius*, *Clevelandia*, *Evermannia*, *Ilypnus*, *Quietula*, *Lepidogobius*, *Gillichthys*, *Typhlogobius*, and *Lethops*, and the northwestern Pacific genera *Gymnogobius*, *Chaenogobius*, *Astrabe*, *Clariger* and *Luciogobiu*s have been identified [29]. Thus, synapomorphies of the Bay Goby clade are: 1) a creased or grooved upper and lower lip making the lip appear double with an infolding or groove on its inside edge; 2) one to five small dorsal bony processes or projections proximally on the inner half of the second (or more) upper pectoral ray(s); 3) glandular mounds, papillae, or raised fleshy edge on the upper and outer surface of the lower limb of the cleithrum under the operculum (possibly present in *Awaous* [62]); 4) lack of ossified teeth on the low rounded intra gill bar rakers posteriorly directed opposite the anteriorly directed gill rakers proper; 5) attachment of proximal end of Beaudelot’s ligment overlaps both basioccipital and first vertebra. The convergent evolution of morphological character suites in these eastern and western Pacific species is presented elsewhere [29].

#### Divergence Times, Paleoclimate, and Landscape Evolution

The habitat of *Eucyclogobius* today depends on a Mediterranean climate system where the lack of summer rain leads to lagoon closure, and the inferred late Miocene (9–15 Mya) divergence of *Eucyclogobius* and *Clevelandia* [10,11,29,30] coincides with development of the California Mediterranean climate system [99]. The sister taxon *Clevelandia* prefers open tidal flat habitat where it associates with invertebrate burrows. Thus, reversed sexual roles [9], more limited reliance on burrows dug by males primarily for reproduction, and the ability to withstand extreme variation in salinity with a preference for lower salinities all likely evolved in *Eucyclogobius* following its divergence from *Clevelandia* and in association with the development of closing lagoon habitat type in a Mediterranean climate [99].

*Eucyclogobiu*s exhibits the most localized population differentiation of any vertebrate on the California coast, with both nuclear and mitochondrial markers supporting multiple clades [10,12,18]. This presumably results from limited dispersal associated with confinement in closing lagoons during the reproductive season. The southernmost population is far more genetically differentiated and is here described as *E*. *kristinae*. Dawson et al. [10], based on typical mitochondrial divergence rates, inferred a divergence time of ~2–4 million years, consistent with our re-analysis of those data. Many recognized species (e.g. Desert Pupfish) diverged in the last glacial cycle, over the last hundred thousand years or even more recently in the transition to the Holocene (discussed in Earl et al. [12]). Thus, *E*. *newberryi* and *E*. *kristinae* diverged over a timescale one to two orders of magnitude greater than these other named species. Additionally, we counted 11 fixed substitutions within a 401-bp fragment of the mitochondrial cytochrome b locus, providing the basis for a DNA barcode distinguishing the two species.

The divergence of the two species of *Eucyclogobius* at approximately 1 Mya is contemporaneous with rapid structural and tectonic evolution of the Southern California Bight and the Los Angeles Basin, the latter of which underwent deformation and uplift in this time period [99]. This includes the emergence of Palos Verdes from the sea for the first time around 1 Mya, and a transition of the Los Angeles Basin from a persistent embayment early in the Pleistocene [103] to an environment that was episodically drained by sea-level low stands over multiple 100,000-year glacial cycles. As a generality, the lagoonal habitats of *Eucyclogobius* were initiated by sea-level rise and the flooding of coastal valleys, and are inferred to have become much more limited during still stand and especially during sea-level fall [12, 104, 105]. Steep coastline exposed at low stand in this region also isolated a number of other estuarine taxa with limited dispersal potential across the Southern California Bight during glacial low stands. The geographic isolation of *E*. *newberryi* and *E*. *kristinae* is consistent with the timing and development of steep coastal features in around the Los Angeles Basin that, in combination with Pleistocene sea-level cycles, likely isolated the two taxa north and south of Los Angeles County over the last 1–2 million years.

### Morphologic differences

#### Sexual Differences and Comparisons

As noted previously, *Eucyclogobius* exhibits a distinct reproductive mode with male brooding of eggs in burrows and female displays [9] associated with dimorphism. In discriminating the species via morphometrics, the combination of sexual dimorphism, limited sample size, allometric growth, and population-level variation within *E*. *newberryi* necessarily complicated the analysis. However, by separating these effects in discriminant analyses, and in generalized linear models for each character (Table 1), we identified morphology clearly associated with sex (Fig 2). These analyses document larger maxillary length, mouth width, and anterior measures of the head in males relative to females (Table 1). These differences appear to result from changes in allometric growth during maturation of males. Our data suggest that this transition occurs in the neighborhood of 20 mm standard length (SL), but further analysis of smaller individuals may shed light on more precise details of this transition. Other more posterior characters that differ between the sexes, such as longer anal fins in males, do not exhibit the same allometric change (Fig 2E). In addition, the larger gap between the caudal fin and dorsal and anal fins (6 SFTDUCB, 10 ANFILCO), and the larger and more consistent space between dorsal fins (35 BETDFINS) in females, may relate to courtship display. The darker second dorsal fin of the female is prominently displayed and undulated in female displays to males [9].

There appears to be some difference in dimorphism between species as determined by the interaction species×sex in the GLMs. One measure, width of the mouth, shows greater dimorphism (larger mouth in males) in *E*. *kristinae* than *E*. *newberryi* (Fig 2D). The limited number of differentially dimorphic characters between species may very well relate to the small number of males of *E*. *kristinae* in the analysis and the resultant limited statistical power. Further examination of the degree of evolution of sexual dimorphism between the two species of *Eucyclogobius* with larger sample sizes is recommended.

#### Ecological Implications

Coherent differences in characters suggest that *Eucyclogobius newberryi* and *E*. *kristinae* differ ecologically. Longer pectoral fins of *E*. *newberryi* are supported by an increased number of fin rays, while increased branched rays of the somewhat larger caudal fin are accompanied by more elongate dorsal and anal fins. These characteristics, in combination with more elongate features of the snout and anterior head, yield a more terminal mouth on a more elongate fish in *E*. *newberryi*. Conversely, in *E*. *kristinae* the greater distance between anterior dorsal fin and posterior jaw, between pelvis and dorsal fin insertion, and between the pectorals ventrally, yield a slightly more robust fish with more upturned mouth. It is also plausible that the larger anal spine in *E*. *kristinae* might play a role in maintaining the body off the substrate. Taken together, these characters suggest a more benthic mode of life in *E*. *kristinae* and more active swimming in *E*. *newberryi*.

Spies and Steele [106] documented higher water temperatures and more rapid larval development in Las Flores, the single *E*. *kristinae* habitat they investigated, relative to the more northern *E*. *newberryi* localities studied. If not simply a byproduct of developmental differences, the reduction in lateral line features in the head of *E*. *kristinae* (which yield a diagnosable difference in neuromast counts) may be associated with reduced need for lateral line function in lower flow regimes and/or a less motile fish. Fish often develop more quickly in warmer water, resulting in lower vertebral and fin ray counts relative to those of the same species developing in cooler water [107]: 536. This may explain the slightly lower counts characterizing *E*. *kristinae*. Further, comparative study of ecological differences between these taxa, such as gut content analysis and controlled behavioral observations in captivity, could expand on and illuminate the nature of these differences.

### Endangered Status

#### Historic Versus Predicted Range

Native American coastal habitation was historically concentrated at lagoons where fresh water met the coastline. In the initial phase of European activity, prior to mechanical pumping and large-scale irrigation infrastructure, the same settings were often subjected to intense manipulation through use of freshwater resources for agriculture and to prevent the incursion of saltwater. By the late 19th century, large coastal lagoon settings at Santa Barbara had been eliminated, and smaller ones—at Las Flores in San Diego County, Dos Pueblos in Santa Barbara County, on the Ventura County coast, in the Salinas/Monterey region, and on Point Reyes—had long been drained, farmed, or tide gated for agricultural or stock use. Farther north in Humboldt Bay, marsh and bay margin habitat was surrounded by levees with occasional tide gates to reclaim and improve marshland for farming, increasingly subdividing Tidewater Goby populations [19,21].

Introduction of exotic fishes into coastal lagoon settings or upstream tributaries was also extensive in many areas. Introduction of carp followed by tens of thousands of young Muskellunge (*Esox masquinongy*) in the late 1800s [108] likely led to extirpation of the Tidewater Goby at Lake Merced on the San Francisco peninsula and perhaps other localities. It is important to keep in mind that the distribution of *Eucyclogobius* was poorly constrained until collections began to increase in the 1940s. There are only eight well-documented collections of Tidewater Goby before 1900, including the original description. From 1900 to 1940, fewer than 25 collections were added to museum records. These four decades saw extensive development of the coast, including oil field development dominating the Los Angeles and Orange County Coasts during the 1920s and 1930s. In the 1930s under the New Deal, federally funded coastal development such as road improvement and flood control channelization of drainages dramatically impacted a large number of lagoonal habitats, especially in San Francisco Bay and south of Point Conception. Tidewater Gobies were first recorded in San Francisco Bay in the 1940s and last observed in 1961, presumably extirpated by continued habitat modification and predation by non-native species. A better understanding of *Eucyclogobius* distribution began with increased sampling after 1940 and was relatively clear by the 1970s. It is in this context of early and ongoing coastal modification and habitat loss, along with delayed surveys of lagoonal habitat, that we should understand the late discovery of *Eucyclogobius* in the range of *E*. *kristinae* and our incomplete understanding of its historic distribution.

Ahnelt et al.’s [15] data on the supraorbital canal clearly show the fish from”Artesian wells in Santa Monica” collected long ago [75] belong to *E*. *newberryi* since the four large specimens have completely developed anterior (and posterior) supraorbital canals. Fish from Aliso Creek, Orange County lack the anterior canals and thus belong to *E*. *kristinae*. Both populations were extirpated before samples suitable for genetic analysis were obtained. Specimens have never been located to support the literature record from San Pedro [36]. Since the extensive rocky shore of Palos Verdes peninsula is north of San Pedro it seems likely that any fish from San Pedro area would have been *E*. *kristinae*. It is reasonable to infer that any historically suitable habitat in this region has since been completely eliminated by urban development, flood channelization, and development of the ports of Los Angeles and Long Beach [105]. The contemporary divide between the two species lies on the Los Angeles Basin, a known barrier to genetic mixing of many marine organisms [109].

To the south in San Diego County, the stretch of coast from the known southern *E*. *kristinae* range limit at Agua Hedionda southward to Los Peñasquitos/La Jolla contains a handful of what were historically closing lagoons that have subsequently been maintained open or otherwise modified [105,110]. Thus, as in the example of San Pedro and the Palos Verdes Peninsula to the north, Los Peñsasquitos and La Jolla provide a southern geomorphic limit that likely confined *E*. *kristinae*. Presence of *Eucyclogobius* in southern California south of Santa Monica was only evident after 1940 as discussed above. By this time, frequent artificial opening of both small and large lagoons was common [110], a practice that severely limits and often precludes the ability of Tidewater Gobies to reproduce. The confirmed range of *E*. *kristinae* resides within, and appears circumscribed by, larger geographic limits to dispersal at the steep Palos Verdes coast to the north and similarly at La Jolla on the south. A similar range gap occurs in the infrequently marine-dispersed Three-spined Stickleback (*Gasterosteus aculeatus*), absent between the San Luis Rey River to the north and the Tijuana River to the south [111]. These two fishes might have either continuously or intermittently occupied more of this intervening range in southern San Diego County prior to human impacts, but were not documented. Future analysis of sediment samples in these coastal systems may provide some evidence of past distributions. Between the 1940s and 1980s, multiple northern Tidewater Goby habitats were discovered in Santa Barbara, San Luis Obispo, and Santa Cruz counties and northward [13]. Several papers have appeared since the 1980s on the status, distribution, and biology of Tidewater Gobies based on both the southern [16] and northern species [9, 19,112–114].

### Endangered Status of *Eucyclogobius*

The state and federal special conservation status designations of *Eucyclogobius newberryi* was based on the loss of many populations then hypothesized, and later confirmed by genetic studies, to be relatively isolated and rarely capable of recolonization over more than 10–15 km of coastline. Both natural and artificial loss of intervening habitat has further separated populations. Individual lagoonal populations of both species face increasing threats of isolation from both natural and anthropogenic events, as well as predation and/or competition from invasive species. One or more of these factors can quickly and unpredictably eliminate a population despite the seasonal presence of large numbers of individuals. Sea-level rise driven by climate change is predicted to further adversely affect both species [115,116]. This combination of threats indicates both species should continue to receive protection until objectives of the Recovery Plan are met [13].

During three years of intense drought from 2013–2015, central and southern California populations of both species have experienced numerous extirpations (see Mapping above; Spies and Jacobs, unpublished). The special conservation status of both species at the state and federal levels should be retained, and care should be taken to preserve overall genetic diversity, as well as the geographically separated and genetically differentiated populations and metapopulation units [12], Thus the description of a southern species does not undercut the significance of the isolated genetic units within *E*. *newberryi*.

#### Critical Endangerment of *E*. *kristinae*

The potential historic range of *E*. *kristinae* suggests a maximum of 150 km of coast from San Pedro to Los Peñasquitos, with an unknown number of populations in closing lagoons and sloughs (Fig 6). The approximately 60-km confirmed range is substantiated only by preserved samples from just 13 localities. This already suggests a high degree of endangerment, but since 1979 Southern Tidewater Gobies have only been recorded at 9 sites over a 29-km range and as noted earlier at just three localities currently. Perhaps most troubling in this declining trend is the inability to recover Southern Tidewater Gobies at Los Flores, the largest system thought to be most persistent and herein designated type locality for *E*. *kristinae*.

Several factors likely contributed to the loss of *E*. *kristinae* habitat. Two large marinas with major jetty structures were constructed in the early 1960s and early 1970s at Oceanside and Dana Point, respectively. These reduced habitat and likely limited longshore dispersal of *E*. *kristinae* and its ability to recolonize beyond the Camp Pendleton range core following population extirpation. On the northern edge of the range, channelization of San Juan Creek combined with Dana Point Marina development have limited lagoon formation at the mouth of San Juan Creek. Aliso Creek, further to the north in Orange County has been filled and developed, is regularly breached to preclude flooding, and now receives increased year-round flow due to residential water use; all of these factors limit the creek’s utility as a Tidewater Goby habitat even if the fish could physically reoccupy the site. In addition to dispersal limitation, lagoons south of Camp Pendleton have been modified to more open tidal systems for a variety of purposes. Agua Hedionda was converted to a tidal lagoon for power plant cooling in 1952. However, somewhat ironically, other lagoons have been converted to tidal systems in the name of “restoration.” Batiquitos, a potential reintroduction site for the Tidewater Goby [13] was converted to a tidal system in the mid-1990s and similar plans are underway to convert Buena Vista lagoon to a tidal system (it is now maintained closed). Other sites have been modified to tidal systems by jetties, or are maintained opened by mechanical means. This approach to “restoration” and lagoon management actually precludes recovery of this endangered species. On Camp Pendleton itself, loss of Tidewater Goby populations at San Mateo Lagoon was likely due to invasive predatory fishes, primarily Green Sunfish, *Lepomis cyanellus*, and Black Bullhead, *Ameiurus melas* (personal observations in the late 1980s, late 1990s, and late 2000s; Swift, Jacobs, Spies). However, the most immediate threat is drought and desiccation of the three small lagoons currently thought to be inhabited, as well as attendant impacts of fires in lagoon drainages. Thus, *E*. *kristinae* appears to be in imminent danger of extinction. Transfer of *E*. *kristinae* to additional localities as mandated in the Recovery Plan [13] is clearly essential at this time, and maintenance of a population in captivity is clearly merited.

## Conclusions

The Southern Tidewater Goby, *Eucyclogobius kristinae*, has a history of genetic isolation (≥ 1 million years) from its sister, *E*. *newberryi*, from which it is separated by a geographic break across the Los Angeles Basin. It can be reliably diagnosed on the basis of meristics—e.g. exposed anterior supraorbital neuromasts on adults (Fig 4, neuromast row no. 1) and higher pectoral fin-ray counts in *E*. *newberryi*—as well as morphometric characters as identified by discriminant function analyses. Sequencing of mitochondrial control region or cytochrome *b* [10] or amplification of any of the suite of microsatellite markers [12] can also provide easy diagnosis, and simple PCR assays for species determination can be easily devised. Morphological distinctions suggest adaptations to a more benthic mode of life in *E*. *kristinae*. Sexual dimorphism associated with an enlarged jaw in adult males is presumptively used in mating burrow construction. Further work to better establish ecological distinction, sexual dimorphism, and/or behavioral differences between the two species is merited. *E*. *kristinae* is critically endangered as it appears to persist in only three sites based on the most recent surveys. Thus, immediate action is needed to prevent extinction of the species during California’s current and persistent drought. All management units of *E*. *newberryi* and *E*. *kristinae* should maintain state and federal endangered status until recovery has been demonstrated.

## Supporting Information

S1 Fig Distribution of *E*. *newberryi* in del Norte and Humboldt Counties. See S1 Text for mapped data.
(TIF)

S2 Fig Distribution of *E*. *newberryi* from Mendocino County to San Mateo County. See S1 Text for mapped data.
(TIF)

S3 Fig Distribution of *E*. *newberryi* from Santa Cruz County to North Santa Barbara County. See S1 Text for mapped data.
(TIF)

S1 Table Measures and counts used in analyses. (XLSX)

S2 Table Size adjusted data for discriminant analyses. (XLSX)

S3 Table Fin ray and vertebral counts from both species of *Eucyclogobius*. (XLSX)

S4 Table Free neuromast and shoulder papillae counts from both species of *Eucyclogobius*. See Fig 2 for location of lines of neuromasts.
(XLSX)

S1 Text Locality information used in mapping. These data are mapped in Fig 6, and S1–S3 Figs.
(DOCX)

S2 Text Synonymy and Valid Literature References. (DOCX)
