# Supplementary figures and images for: A New Species of the Bay Goby Genus Eucyclogobius, Endemic to Southern California: Evolution, Conservation, and Decline
Source: PLoS One. 2016 Jul 27;11(7):e0158543. doi: 10.1371/journal.pone.0158543 (PMC4963035; doi:10.1371/journal.pone.0158543)

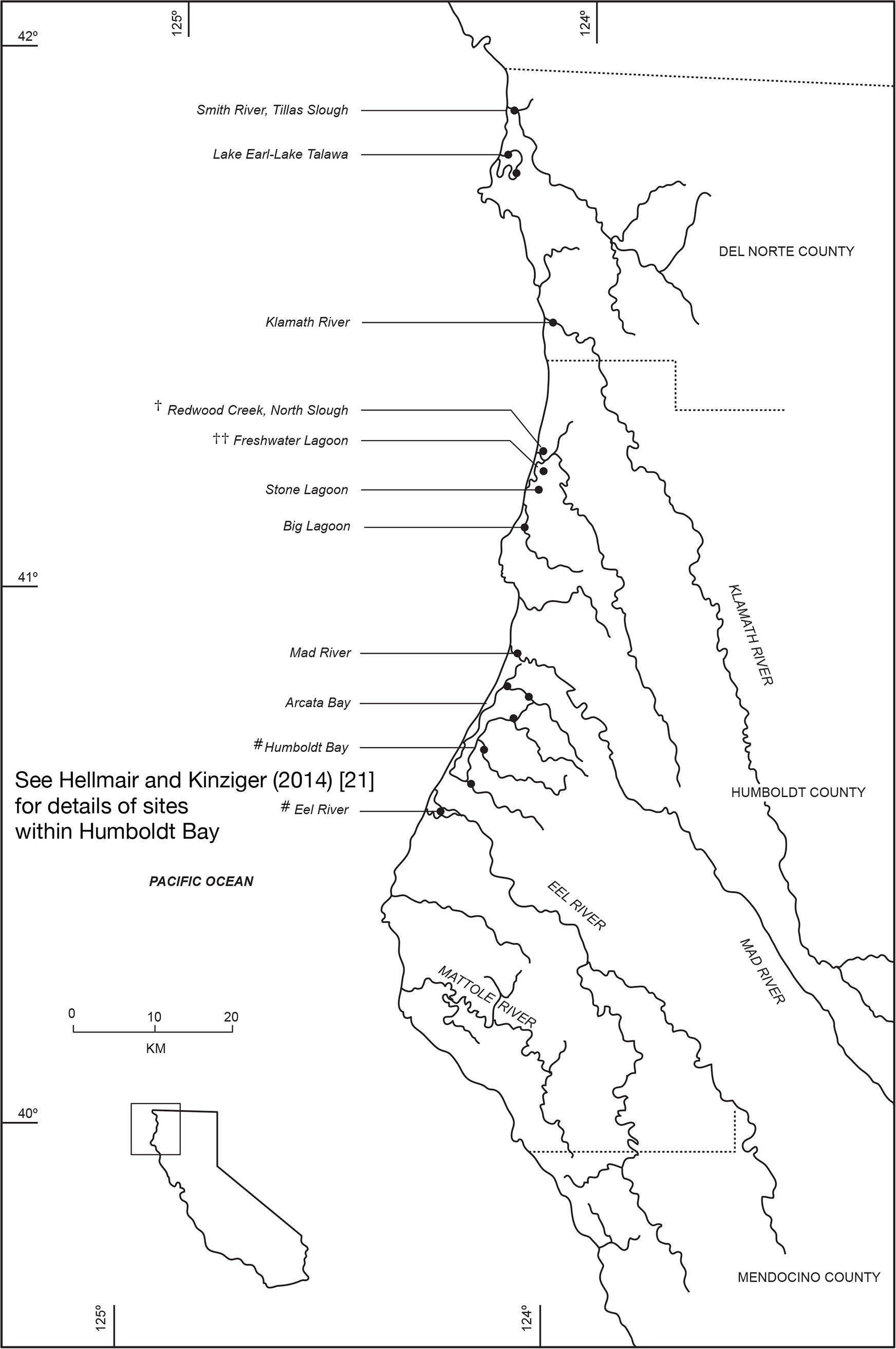

Supplement: S1 Fig — See S1 Text for mapped data. (TIF) [file pone.0158543.s001.tif]

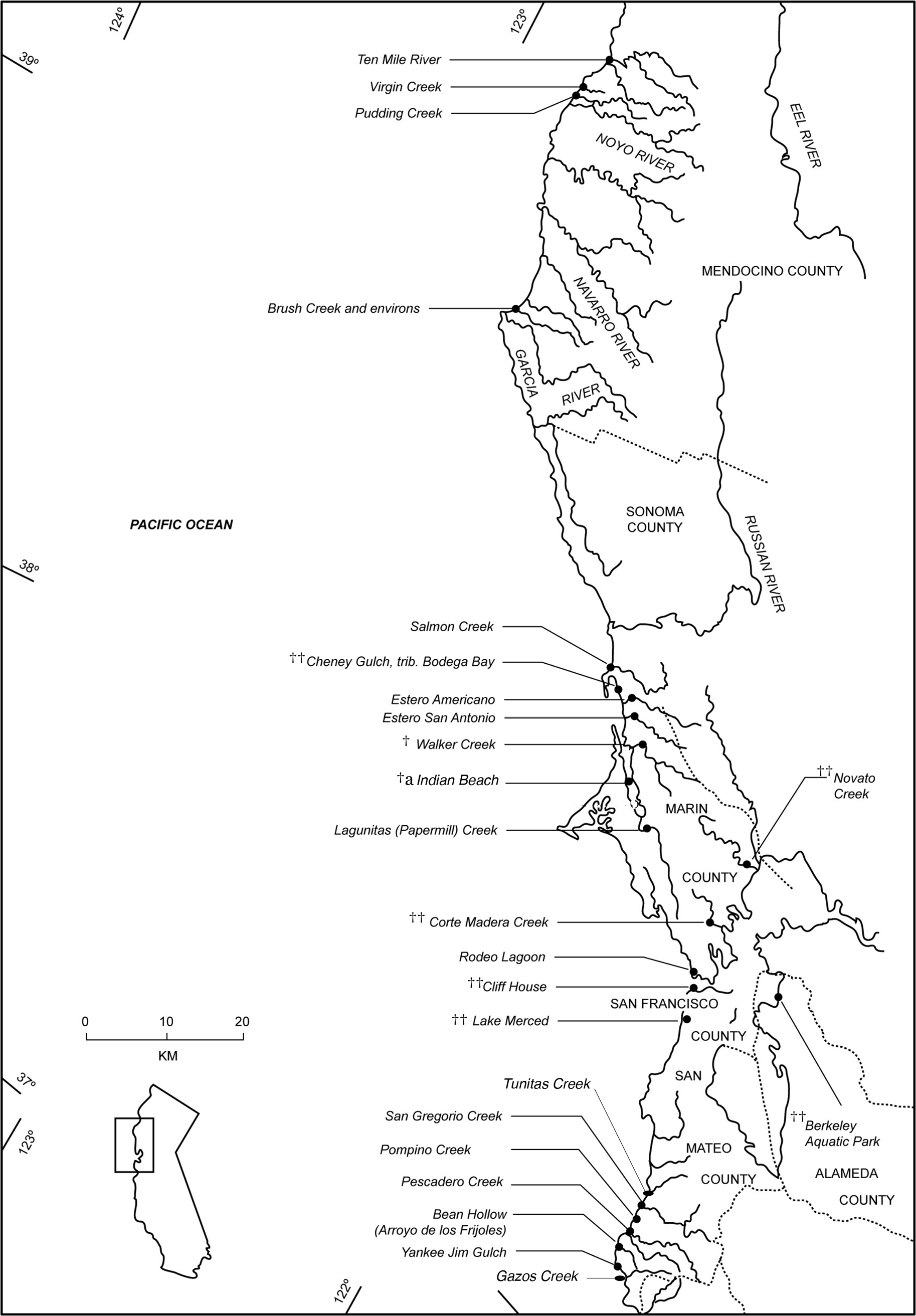

Supplement: S2 Fig — See S1 Text for mapped data. (TIF) [file pone.0158543.s002.tif]

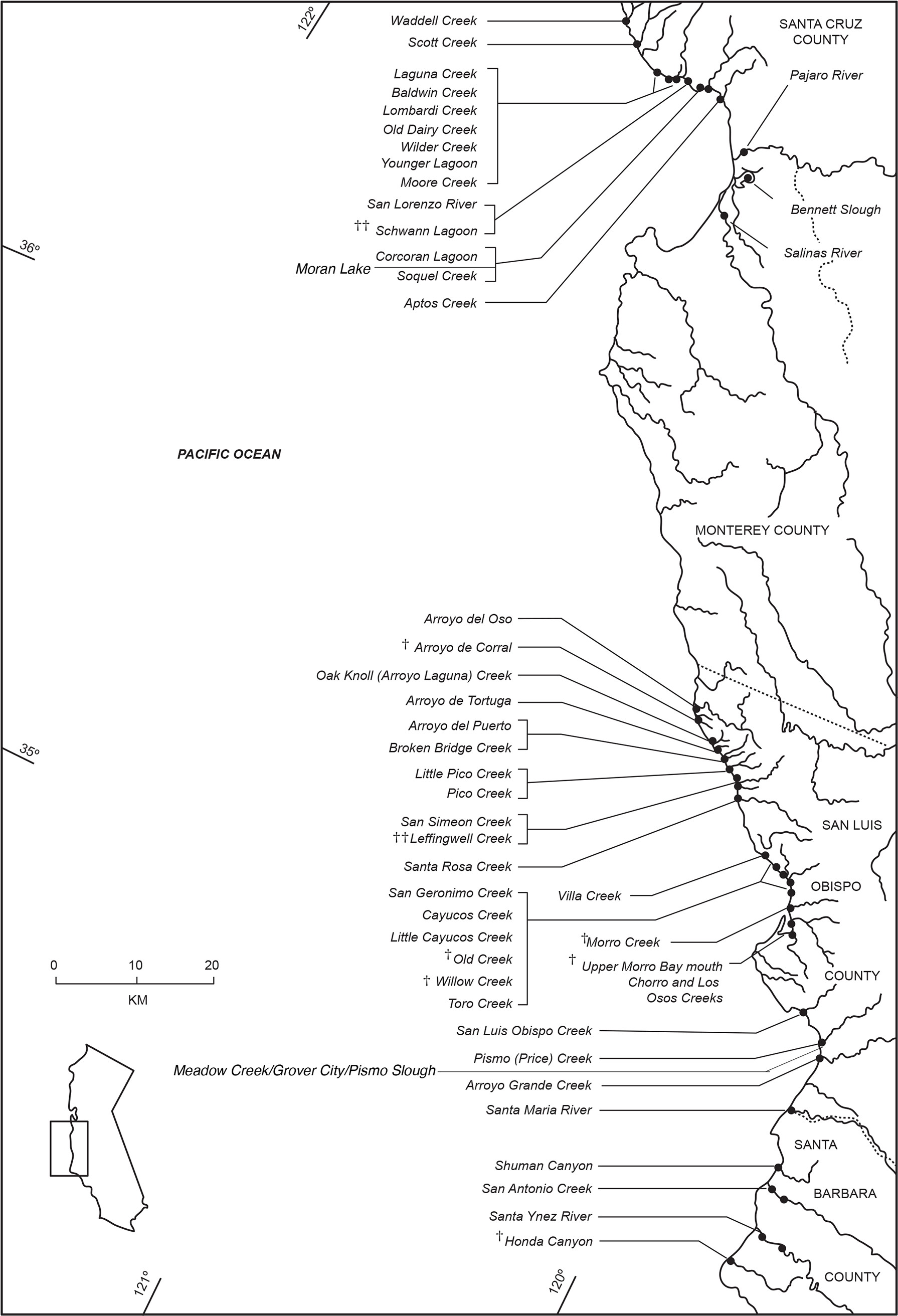

Supplement: S3 Fig — See S1 Text for mapped data. (TIF) [file pone.0158543.s003.tif]
