# Supplementary material for: A New Species of the Bay Goby Genus Eucyclogobius, Endemic to Southern California: Evolution, Conservation, and Decline
Source: PLoS One. 2016 Jul 27;11(7):e0158543. doi: 10.1371/journal.pone.0158543 (PMC4963035; doi:10.1371/journal.pone.0158543)
Supplement: S1 Text — These data are mapped in Fig 6, and S1–S3 Figs. (DOCX) [file pone.0158543.s008.docx]

S1 Text – Locality In formation used in mapping (Figue 6, S1-3 Figures)

Museum numbers LACM unless otherwise indicated and numbers not repeated if listed above for specimens examined. Vouchers in the Jacobs Laboratory, University of California, Los Angeles, DJ UCLA (collection numbers BTS...Brenton Spies, CCS... Camm Swift, collection permits respectively). Visually recorded by Camm C. Swift, Douglas Reischbeiter, California State Parks Department, Jerry Smith, San Jose State University, or Andrew Kinziger, Humboldt State University, indicated by DJ UCLA, CCS, DR, JS SJSU, and AK HSU respectively. As noted above, some sites have not been continuously occupied, some have been extirpated and there have been a handful of efforts at reintroduction. † marks localities where fish were absent in surveys in the last two years and the fish is likely currently extirpated. †† marks localities that may not have been recently surveyed but where habitats have been modified such that conditions typical of Tidewater Goby habitat are no longer present. * indicates systems that have not been recently sampled. ^#^ indicates multiple discrete localities are present within a larger system. ^e^ indicates a locality where presence was determined by environmental DNA only. ^a^ denotes location of an attempted artificial reintroduction.

*Eucyclogobius newberryi*: **Del Norte County:** Tillas Slough, Smith River (CAS 27187); Lake Earl-Lake Tolowa (42669-1); Klamath River, Molly Schmelzle, late 2014, AK HSU); **Humboldt County:** †Redwood Creek (Terry Hofstra, Tim Salamunovich, personal communication, September, 1989); ††Freshwater Lagoon (CAS Acc. 1952:x:30); Stone Lagoon (35342-3); Big Lagoon (35341-1); Mad River (Darren Ward, mid-February 2015, AK HSU); ^#^ Arcata Bay (northern Humboldt Bay) (35335-5; HSU 2845); southern ^#^Humboldt Bay-Elk River (HSU 4953, 4948, see; ^#^ Eel River (HSU 3655); **Mendocino County:** Ten Mile River (35331-1); Virgin Creek (35329-2); Pudding Creek (35328-1); Brush Creek (42666-2); ^e^Caspar Creek (Molly Schmelzle, late 2014, AK HSU); **Sonoma County:** Salmon Creek (35317-3); ††Cheney Gulch (Bodega Harbor) (CAS 25512); **Sonoma-Marin county line:** Estero Americano (37380-1); **Marin County:** Estero San Antonio (42665-2); †Walker Creek (USNM 067297); mid-Tomales Bay (^a^ †Indian Beach lagoon, 2009-2012, Darren Fong, Golden Gate National Seashore); upper Tomales Bay-lower Lagunitas (Papermill)-Tomasini creeks (CAS [ex. UCLA 263-9-1-A); **Marin County:** Rodeo Lagoon (CAS Acc. 1969:vii:16); ††Corte Madera Creek (CAS 23685); ††Novato Creek (CAS 12995); **Alameda County:** ††Aquatic Park, mouth Strawberry Creek (CAS Acc. 1964:xi:13); **San Francisco County:** ††Cliff House (NMW 31182); ††Lake Merced (CAS 12483); **San Mateo County:** Tunitas Creek (DJ UCLA BTS 15-20); San Gregorio Creek (35297-2); Pompinio Creek (DJ UCLA- BTS 14 23), Pescadero Creek (CAS 86281); Bean Hollow (Arroyo Frijoles) (35293-2); Yankee Jim Canyon (RB, fall, 2013); Gazos Creek (DJ UCLA, BTS 14-27); **Santa Cruz County**, Waddell Creek (CAS 28670); Scott Creek (CAS 20896); Laguna Creek (35286-2); Baldwin Creek (35284-1); Lombardi Creek (DJ UCLA, BTS 15-032); Old Dairy Creek (DJ UCLA, BTS 15-33); Wilder Creek (called Meder Creek in [16]) (35285-2); Younger Lagoon (56191-1); Moore Creek (43763-1); San Lorenzo River (56204-1); †† Schwann Lagoon (called Twin Lakes-Woods Lagoon in [16]) (35281-2); Corcoran Lagoon (called the Rodeo Gulch in [16]) (35279-1); Moran Lake (DJ UCLA, BTS 14-36); Soquel Creek (DJ UCLA, BTS 14-37); Aptos Creek (37577-2); **Santa Cruz-Monterey County line:** Pajaro River (CAS 31894); **Monterey County:** Bennett Slough (42656-2); Salinas River (CAS 48290); **San Luis Obispo County:** Arroyo del Oso (35257-2); †Arroyo de Corral (42349-1); Oak Knoll Creek (Arroyo Laguna) (36195-6); Arroyo de Tortuga (“unnamed canyon 2.2 km NW of San Simeon Pt. in [16]) (36663-2); Arroyo del Puerto (42351-1); Broken Bridge Creek (42352-1); Little Pico Creek (36666-2); Pico Creek (SIO72-88); San Simeon Creek (SIO72-87); ††Leffingwell Creek (42353-1); Santa Rosa Creek (36667-1); Villa Creek (42355-1); San Geronimo Creek (DJ UCLA, CCS 08-020); Cayucos Creek (36669-1); Little Cayucos Creek (DJ UCLA, CCS 08-022); †Old Creek (36670-2); †Willow Creek (DJ UCLA, BTS 15-061); Toro Creek (DJ UCLA, BTS 14-93); †Morro Creek (FMNH 9121); †upper Morro Bay, lower Chorro, Los Osos creeks (35573-1, 42348-2); San Luis Obispo Creek (CAS SU 653); Pismo (Price) Creek (36673-3); Pismo Slough, opposite Grover City, Also Meadow Creek in more recent collections (SIO 51-10); Arroyo Grande Creek (DJ UCLA, CCS 08-029); **San Luis Obispo-Santa Barbara county line**, Santa Maria River (42345-1); **Santa Barbara County**: Shuman Lagoon (36197-1); San Antonio Creek (36200-1; 39966-1; UCLA W82-2); Santa Ynez River (42343-1); †Honda Canyon (Swift, 16 September 1995); Jalama Creek (31425-2); *Damsite Canyon (56577-1); *Cañada del Cojo (56576-1); *Arroyo San Augustin (SBMNH 00015); *Cañada de las Agujas (SBMNH 00957); *Arroyo El Bulito (36658-1); *Cañada del Agua (1981); *Cañada de Santa Anita (SBMNH 00959); *Cañada de Alegria (SBMNH 00961); *Cañada de Augua Caliente (42340-1); Gaviota Creek (1693); †Arroyo Hondo (UMMZ 130666); †Arroyo Quemado (36193-1); Refugio Creek (DJ UCLA, CCS 05 089); †Tecolote Canyon (42339-1); †Winchester (Bell, Ellwood) Canyon (SBMNH 1077); †Devereaux Slough (SBMNH 00592); †University of California, Santa Barbara, Campus Lagoon (SBMNH 0592); Tecolotito Creek (trib. Goleta Slough) (DJ UCLA), Arroyo Burro (SBMNH 1407); Mission Creek (DJ UCLA, CCS 02 025); Laguna Creek (DJ UCLA, CCS 02 026); †Sycamore Creek (DJ UCLA,CCS 02 027); Andre Clark Bird Refuge (DJ UCLA, CCS 02 028); Arroyo Paredon (DJ UCLA, CCS 02 090); El Estero, Carpinteria Salt Marsh (UMMZ 63285); Carpinteria Creek (UMMZ 133067); **Santa Barbara-Ventura county line:** Rincon Creek (56370-1); **Ventura County:** Ventura River (36216-1); †Santa Clara River (34071-1); Ormond Lagoon, (“J” Street Drain, Oxnard Drain) (56214-1); †Calleguas Creek, tributary of Mugu Lagoon (UMMZ 133072); † (Big) Sycamore Canyon (CCS 08); **Los Angeles County:** ^a^ Malibu Creek (UCLA(CAS) W55-272); Topanga Creek (56274-1); ††Artesian Wells, Santa Monica (NMW 33915).

*Eucyclogobius kristinae*: **Orange County:** ††Aliso Creek (42372-1); ††San Juan Creek (UMMZ 131650); **San Diego County:** † ^a^ San Mateo Creek (36189-2); San Onofre Creek (42692-2); †Las Flores (Las Pulgas) Creek (42691-2); Hidden Lagoon (DJ UCLA, CCS 98-116); †Aliso Canyon (DJ UCLA, CCS 98-117); †French Canyon (CCS); Cockleburr Canyon (DJ UCLA, CCS 98-119); †Santa Margarita River (36191-3); †San Luis Rey River (UCLA W58-1); ††Buena Vista Lagoon (UCLA W53-235); ††Agua Hedionda Lagoon (UMMZ 131809).
