# Supplementary material for: A New Species of the Bay Goby Genus Eucyclogobius, Endemic to Southern California: Evolution, Conservation, and Decline
Source: PLoS One. 2016 Jul 27;11(7):e0158543. doi: 10.1371/journal.pone.0158543 (PMC4963035; doi:10.1371/journal.pone.0158543)
Supplement: S2 Text — (DOCX) [file pone.0158543.s009.docx]

Complete synonymy and references for the genus *Eucyclogobius,* and species *E. newberryi, and E. kristinae*

Synonymy and Valid Literature References

Genus *Eucyclogobius* Gill, 1862

*Eucyclogobius* Gill 1862:[1]: 279, 330, 332, brief original description, type species *Gobius newberrii* Girard 1856:[2]: 136, by original designation and also monotypic; Gill, 1863:[3]: 264, 265, list; Bleeker 1874:[4]: 298, 319, *Gobius newberrii* designated as genotype, after Gill; Jordan and Gilbert 1882:[5]: 331, squamation compared to *Gobius* (now *Microgobius*) *emblematicus*; Jordan and Gilbert 1883[6]: 637-638, subgenus of *Lepidogobius;* Jordan, 1887:[7]: 894, list, subgenus of *Lepidogobius*; Jordan and Eigenmann 1887:[8]: 517, subgenus of *Lepidogobius*; Eigenmann and Eigenmann 1888:[9]: 69, listed, subgenus of *Lepidogobius*; Jordan and Evermann 1896:[10]: 459, list; Jordan and Evermann 1898:[11]: 2248, description, comparison with *Lepiogobius*; Jordan 1919:[12]: 316, list; Jordan 1923:[13]: 225, classification; Hubbs 1926:[14]: 3, characteristics, distinct from *Lepidogobius*; Jordan et al. 1930:[15]: 444, list; Shapovalov 1941:[16]: 441, California list; Barlow 1961:[17]: 424, related to other northeastern Pacific estuarine gobies; Golvan 1962:[18]: 148, list; Norman 1966:[19]: 405, 415, key to world goby genera, list; Nelson 1976:[20]: 278, representative gobiid genus; Birdsong et al. 1988:[21]: 187, axial skeleton, gobiid classification; Eschmeyer and Bailey 1990:[22]: 144-145, catalogue of genera of recent fishes; Eschmeyer 1990:[23]: 488, list in classification; Burr and Mayden 1992:[24]: 23, list of North American freshwater genera; Pezold 1993:[25]: 640, classified in subfamily Gobionellinae of family Gobiidae; Nelson 1994:[26]: 416, in subfamily Gobionellinae of Gobiidae; Larson 2001:[27]: 4, phylogenetic relationships; Thacker 2003:[28]: 357, 359, 360-362, molecular phylogeny; Nelson et al. 2004:[29]: 171, list; Nelson 2006:[30]: 423, in subfamily Gobionellinae of Gobiidae; Kindermann et al. 2007:[31]: 13-56, osteology; Thacker 2009:[32]: 96, molecular phylogeny, in family Gobionellidae; Ruber and Agorreta 2011:[33]: 31, 40-41, microsatellite studies, gobiid molecular phylogenies; Pezold 2011:[34]: 94, list, north Pacific gobionellid genera; Van Tassell 2011:[35]: 143, list, Gobiformes of the Americas; Thacker 2013:[36]: 371, member of North Pacific group; Tornabene et al. 2013:[37]: 4, 11, relationships; Agoretta et al. 2013:[38]:627, phylogenetic relationships; Ellingson et al. 2014:[39]: 465, 467, 472-475; phylogeny, morphological convergence with western Pacific species.

*Eucyclogobius newberryi* (Girard, 1856)

Northern Tidewater Goby

*Gobius newberryi* Girard, 1856:[2]: 136, original description as “…a brief account, extracted from final reports…;” Girard 1857:[40]: 539-541, figures 5-8, plate 25, misspelled as “*newberrii,”* description, illustration, specimens from Tomales Bay; Girard 1858:1859:[41]: 128, [repeated] descriptions in editions of the western railroad surveys, types USNM 360, 24 specimens from Tomales Bay; Gunther 1861:[42]: 77, list, description from Girard, as “*newberrii*”; Steindachner 1879:[43]: 135-137, description of specimens from artesian springs, Santa Monica, California, as “*newberrii*”.

*Lepidogobius newberryi*, Gill 1859:[44]: 14, list; Lockington 1880:[45]: 18, list of California fishes; Jordan and Gilbert 1881:[46]: 455, list, table of Pacific coast fishes; Jordan and Gilbert 1883:[6]: 637-638, description, range, comparisons; Jordan 1887:[7]: 894, list, included within subdivision or subgenus *Eucyclogobius*; Jordan and Eigenmann 1887:[8]: 502-503, 515, 517, keys, description, list; Eigenmann and Eigenmann 1888:[9]: 69, list, as “*newberrii*”; Eigenmann and Eigenmann 1892:[47]: California list, footnote record for San Pedro, no extant specimens known; Ginsburg 1945:[48]: 134-137, 139, caudal fin ray counts.

*Eucyclogobius newberryi,* Gill 1863:[3]: 264-265, synonymy; Bleeker 1874:[4]: 298, 319, classification; Jordan and Gilbert 1881:[46]: 53, listed; Jordan and Gilbert 1882:[5]: 331, squamation compared to *Gobius* (now *Microgobius*) *emblematicus*; Jordan 1895:[49]: 141-142, San Luis Obispo Creek; Jordan and Evermann 1896:[10]: 459, list; Jordan and Evermann 1898:[11]: 2248, description, range, confined to freshwater; Hubbs 1921:[50]: 5, central California records; Hubbs 1926:[51]: 3, range, comparison with *Lepidogobius lepidus*; Ulrey and Greeley 1928:[52]: list; Ulrey 1929:[53]: 10, list; Evermann and Clark 1931:[54]: 58, 63, California habitat, list; Barnhart 1936:[55]: 82, description, habitat, range; Schrenkeisen 1938:[56]: 274, description, biology, range; Dill and Shapovalov 1939:[57]: 322, aquarium fish potential; Needham 1940:[58]: 180-182, diet in Waddell lagoon; Shapovalov 1943:[59]: 445, California freshwater; Hubbs 1947:[60]: 147-148, lower Salinas River records; Shapovalov and Dill 1950:[61]: 387, California list; Shapovalov and Taft 1954:[62]: 26-27, 257, 277, biology in Waddell Creek; Eddy 1957:[63]: 230, list, illustration, freshwater of California; Shapovalov et al. 1959:[64]: 174, California list; Kimsey and Fisk 1960:[65]: 474, key California freshwater fishes; Kimsey and Fisk 1964:[66]: 53, related to *Gillichthys mirabilis*; Bailey et al. 1960,[66] 35, list; Norman 1966:[19]: 405, 415, type species of genus; Eddy 1969:[68]: 266, listed, freshwater in California; Hubbs and Miller 1965:[69]: 44, associated with rainwater killifish, *Lucania parva*, introduced in San Francisco Bay about 1959; Bailey et al. 1970:[70]: 51, list; Miller and Lea 1972:[71]: 186-187, key, characters, range; Eldridge and Bryan 1972:[72]: 4, larvae rare in Humboldt Bay; Fierstine et al. 1973:[73]: 84, localities in upper Morro Bay; Miller and Lea 1976:[74]: 240, range extended; Moyle 1976:[75]: 85, 344-346, key, biology, distribution; Shino 1976:[76]: list, common name; Eddy and Underhill 1978:[77]: 194, freshwater in California; Hubbs et al. 1979:[78]: 44, California list; Deacon et al. 1979:[79]: 40, species of Special Concern, California; Swift 1980:[80]: 788, biology, distribution map; Robins et al. 1980:[81]: 55, list; Gotshall et al. 1980:[82]: 227, list, Humboldt Bay; Ono et al. 1983:[83]: 242, list of endangered fish; Eschmeyer et al. 1983:[84]: 262, pl. 19, illustration, characters, range; Leidy 1984:[85]: 9, 25-26, 73, 171, historical occurrence, San Francisco Bay; McGinnis 1984:[86]: 60, 232, illustration, biology; Minckley et al. 1986:[87]: 551, 574, 587, zoogeography; Swift et al. 1989:[88]: 1-19, biology, distribution, conservation status; Williams et al. 1989:[89]: 12, conservation status; Moyle and Williams 1990:[90]: 280-281, decline in California; Eschmeyer and Bailey 1990:[22]: 144-145, type of genus; Swift 1990:[91]: 4-5, habitat, biology, popular account; Robins et al. 1991:[92]: 64, list; Page and Burr 1991:[93]: 3, list, marine invader of freshwater; Yoklavich et al. 1991:[94]: 473, occurrence in Elkhorn Slough; Burr and Mayden 1992:[24]: 19, 56, brackish species; Mayden et al. 1992:[95]: list, conservation status; Monaco et al. 1992:[96]: 259-260, 262, U.S. west coast estuarine fish assemblages; Chamberlain and Barnhart 1993:[97]: 775, 777, 779, 781, habitat restoration, Humboldt Bay; Swenson 1993:[98] 441: marshes significant habitat; Swift et al. 1993:[99]: 129-130, status, conservation, southern, central California; Brown and Swenson 1994:[100]: 390-391, popular account of endangered species; Swenson and McCray 1996:[101]: 956-970, feeding ecology; Watson 1996:[102]: 1215-1219, larval description; Lafferty et al. 1996:[103]: 254, endangered status; Love 1996:[104]: 301-303, characters, biology, conservation; Swenson 1997:[105]: 27-40, sex-role reversal; Capelli 1997:[106]: 1-18, habitat management; Lafferty and Page 1997:[107]: 589-592, predation by clawed frog; Lafferty et al. 1999a:[108]: recolonization, metapopulation dynamics; Lafferty et al. 1999b:[109]: dispersal, recolonization; Swenson 1999:[110]: biology, conservation, behavior, distribution; Fuller et al. 1999:[111]: 459, 464, threats of non-native gobies; Ambrose and Meffert 1999:[112]: 331-336, 338, part of fish community in Malibu Lagoon; Musick et al. 2000:[113]: 18, 21, 23-24, extinction risk; Moyle and Davis 2000:[114]: 257, list; Matern 2001:[115]: 597, impact of introduced shimofuri goby; Mendoca et al. 2001:[116]: 91-95, microsatellite variation, central California; Dawson et al. 2001:[117]: 1167-1179, range-wide phylogeography; Swift et al. 2002:[118]: 7-9, biology, status as resource; Moyle 2002:[119]: 3, 10, 19, 93, 430-434, key, habitat, biology, conservation; Dawson et al. 2002:[120]: 1065-1075, phylogeography compared to *Clevelandia ios*; Moeller et al. 2003:[121]: 425, pollutants in fish, Malibu Creek; Anhelt et al. 2004:[122]: 385-398, variation in head canal development; Marchetti et al. 2004:[123]: 1514, 1525, effect of invasive species; Jacobs et al. 2004:[124]: 614, 626, 634-636, 640, divergence times, speciation mechanisms; Nelson et al. 2004:[29]: 171, North American list; U.S. Fish and Wildlife Service 2005:[125]: 1-77, endangered species recovery plan; Leidy et al. 2005:[126]: 219, 236, associate of coho salmon, San Francisco Bay tributaries; Swift 2006:[127]: 625, distribution; Barton 2006:[128]: 842, conservation; McGinnis 2006:[129]: 85, 357-358, illustration, biology, characters, range; Horn et al. 2006:[130]: 18, distribution, zoogeography; Dawson et al. 2006,[131] 34, 38-39, phylogeography; Allen et al. 2006:[132]: 126, 128-130, 132,134, ecology in estuaries; Chamberlain 2006:[133]: 2-33, northern California distribution, habitat; McGourty et al. 2007:[134]: 665-660, microsporidian parasite; McGourty et al. 2008:[135]: 18-32, reproduction, habitat, parasites, northern California; Frimodig and Goldsmith 2008:[136]: 194-199, parasites, Humboldt Bay localities; Jelks et al. 2008:[137]: 407, conservation status; Earl et al. 2010:[138]: 103-114, phylogeography, distinctness of southern population; McCraney et al. 2010:[139]: genetic drift in fragmented northern populations; Love 2011:[140]: 496-497, photo, characters, biology; Ruber and Agorreta 2011:[33]: 40-41, in part, reanalysis of molecular phylogeny of gobiids; Van Tassell 2011:[35]: 143, in part, list of Gobiiformes of the Americas, distribution, habitat; Borges et al. 2011:[141]: 414, in part, early life history characteristics; U. S. Fish and Wildlife Service 2013:[142]: 8746-8819, in part, designation of Critical Habitat under the Endangered Species Act; Page et al. 2013:[143]: 176, North American list; Thacker 2013:[36]: 372, 374, 376, 377, phylogeny, characters within Gobionellidae; Torrnabene et al. 2013:[37]: 1, 11, gobiid phylogeny; Dawson 2014:[144]: 56, phylogeography; Spies et al. 2014:[145]: 165-175, duration, growth of larval stage; Richmond et al. 2015:[146]: 16, genetics in Ventura and Santa Clara rivers; Hellmair and Kinziger 2014:[147]: extinction potential, northern California; Martin 2014:[148]: 593, air breathing during hypoxic conditions; Collins and Melack 2014:[149]: 103-106, 110, habitat in Devereux Slough; Swift et al. 2015:[150]: 162, potential predation by Mississippi silversides; Kinziger et al. 2015:[151]: 1-10, extinction colonization dynamics, northern California; Martin 2015:[152]: 46, 91, air breathing; Chase and Todgham 2016:[153]: 94-107, laboratory effects on growth of co-existing species; Schmelzle and Kinziger 2016:[154]: detecting habitat occupancy with e-DNA; Spies and Steele 2016:[155]: 243-255, effects of temperature and latitude on larval traits.

*Gillichthys mirabilis*, Starks and Morris 1907:[156]: 226-227, misidentification as “Specimens examined from Mendocino County, 1¾” in length, contained mature eggs.”, Mendocino County record; Snyder 1938:[157]: 358, misidentification, Waddell Creek lagoon.

*Eucyclogobius* (N), Ellingson et al. 2014:[39]: 472, morphological convergence with western Pacific species of *Gymnogobius*.

*Eucyclogobius kristinae* new species, Swift, Spies, Ellingson & Jacobs, new species

Southern Tidewater Goby

*Eucyclogobius kristinae****,*** Swift, Spies, Ellingson, and Jacobs***.*** urn:lsid:zoobank.org:act:CC641CE5-7DAE-466E-BA3F-D23994068F2B.

*Gillichthys mirabilis*, Metz 1912:[158]: 41, misidentification, record from Aliso Creek, Laguna Beach, Orange County.

*Eucyclogobius newberryi*, Miller 1939:[159]: 41-48 and 1943:[160]: 168, records from San Juan Creek, Orange County; Swift et al. 1989:[88]: 1-19, in part, biology, distribution, illustration; Goldberg 1977:[161]: 557-559, ovarian cycling; Ruber and Agoretta 2011:[33], in part, reanalysis of gobiid molecular phylogeny; Van Tassell 2011:[35]: 143, in part, list of Gobiiformes of the Americas; Dawson et al. 2001:[117]: 1167-1179, range-wide phylogeography; Dawson et al. 2002:[120]: 1065-1075, phylogeography compared to *Clevelandia ios*; Anhelt et al. 2004:[123]: 385-398, variation in head canal development; Earl et al. 2010,[136] 103-114, phylogeography, distinctness of southern population; Borges et al. 2011:[141]: 414, in part, early life history characteristics; Spies et al. 2014:[143]: 165-175, duration, growth of larval stage; Spies and Steele 2016:[153]: 243-255, effects of temperature and latitude on larval traits.

*Eucyclogobius* (S), Ellingson et al. 2014:[39]: 472, convergence with western Pacific species.

References for Synonymies only

**1.** Gill TN. Notice of a collection of the fishes of California presented to the Smithsonian Institution by Mr. Samuel Hubbard. Proc Acad Nat Sci Phila. 1862;14(6):274-282.

**2.** Girard C. Contributions to the ichthyology of the western coast of the United States from specimens in the museum of the Smithsonian Institution. Proc Acad Nat Sci Phila. 1856;8(3):131-137.

**3.** Gill TN. Descriptions of the gobioid genera of the western coast of temperate North America. Proc. Acad Nat Sci Phila. 1863;15(5):262-267.

**4.** Bleeker P. Esquisse d’un systѐme naturelles des Gobioides. Arch Neerl Sci Nat Haarlem. 1874;9:289-331.

**5.** Jordan DS, Gilbert CH. Descriptions of nineteen new species of fishes from the Bay of Panama. Bull US Fish Commn. 1882;1:306-335.

**6.** Jordan DS, Gilbert CH. Synopsis of the fishes of North America. Bull US Nat Mus. 1883;16:1-1018 pp. 52.

**7.** Jordan DS. XXIV. A catalogue of the fishes known to inhabit the waters of North America, north of the Tropic of Cancer, with notes on the species discovered in 1883 and 1884. Rep Comm, US Comm Fish and Fisheries. 1887;12:789-973.

**8.** Jordan DS, Eigenmann CH. A review of the Gobiidae of North America. Proc US Nat Mus. 1887;9(587):477-518.

**9.** Eigenmann CH, Eigenmann RS. A list of American species of Gobiidae and Callionymidae, with notes on the specimens contained in the Museum of Comparative Zoology, at Cambridge, Massachusetts. Proc Calif Acad Sci. 1888; Ser 2,1(1):51-78.

**10.** Jordan DS, Evermann BW. A check-list of the fishes and fish-like vertebrates of North and Middle America. Rep US Fish Comm. 1896;21, Appendix 5:207-584.

**11.** Jordan DS, Evermann BW. The fishes of North and Middle America: a descriptive catalogue of the species of fish-like vertebrates found in the waters of North America, north of the Isthmus of Panama. Bull US Natl Mus. 1898;47(3):2183-3136.

**12.** Jordan DS. The genera of fishes, Part III, from Guenther to Gill, 1859-1880, twenty-two years, with the accepted type of each. A contribution to the stability of scientific nomenclature. Leland Stanford Junior University Publications, University Series. 1919;285-410.

**13.** Jordan DS. A classification of fishes including families and genera as far as known. Stanford University Publications, University Series, Biological Sciences. 1923;3(2):77-243.

**14.** Hubbs CL. Notes on the gobioid fishes of California, with descriptions of two new genera. Occ Pap Mus Zool Univ Mich. 1926;(169):1-6.

**15.** Jordan DS, Evermann BW, Clark HW. Check list of the fishes and fishlike vertebrates of North and Middle America north of the northern boundary of Venezuela and Columbia 1930. Rep US Comm Fish, Fiscal Year 1928, Part II, No. 1055. US Govt Print Off.

**16.** Shapovalov L. The freshwater fish fauna of California. Sixth Pacific Science Conference, proceedings. 1943;441-446.

**17.** Barlow GW. Gobies of the genus *Gillichthys*, with comments on the sensory canals as a taxonomic tool. Copeia. 1961;No.4:423-437.

**18.** Golvan Y–J. Catalogue systématique des noms de genres de poisons actuels de la X^e^ édtion du « Systema naturae » de Charles LINNE jusqu’à la fin de l’année 1959. Ann Parasit hum comp. 1962;37(6)(Suppl):1-227. [Reprint 1965].

**19.** Norman JR. A draft synopsis of the orders, families, and genera of recent fishes and fish-like vertebrates (excluding Ostariophysi, Scleroparei, Amodytidae, and a few other families, notably Centrarchidae, Percidae, and Cichlidae). Trustee of the British Museum (Natural History), London, UK. 1966.

**20.** Nelson JS. Fishes of the World. John Wiley and Sons, New York, NY. 1976.

**21.** Birdsong RS, Murdy EO, and Pezold FL. A study of the vertebral column and median fin osteology in gobioid fishes with comments on gobioid relationships. Bull Mar Sci. 1988;42(2):174-214.

**22.** Eschmeyer WN, Bailey RM. Part I. Genera of Recent Fishes. In: William N. Eschmeyer WN. Catalog of the Genera of Recent Fishes. California Academy of Sciences, San Francisco, CA. 1990; pp. 7-433.

**23.** Eschmeyer WN. Part II. Genera in a Classification. In: William N. Eschmeyer. Catalog of the Genera of Recent Fishes. California Academy of Sciences, San Francisco, CA. 1990; pp. 435-501.

**24.** Burr BM, Mayden RL. Chapter 2. Phylogenetics and North American freshwater fishes. Pp. 18-75. In: Mayden, RL, editor. Systematics, historical ecology, and North American freshwater fishes. Stanford University Press, Stanford, CA. 1992.

**25.** Pezold F. Evidence for a monophyletic Gobiinae. Copeia. 1993;634-643.

**26.** Nelson JS. Fishes of the World. 3^rd^ Edition. John Wiley and Sons, Inc., New York, NY. 1994.

**27.** Larson H. A revision of the gobiid fish genus *Mugilogobius* (Teleostei: Goboiodei: Gobiidae) and its systematic placement. Rec West Aust Mus. 2001; Suppl 62:1-233.

**28**. Thacker C. Molecular phylogeny of the gobioid fishes. Mol Phylo Evol. 2003;26:354-368.

**29.** Nelson JS, Crossman EJ, Espinosa Pérez H, Findley LT, Gilbert CR, Lea RN, et al. Common and scientific names of fishes from the United States, Canada, and Mexico. Sixth Edition. American Fisheries Society, Bethesda, MD, 2004. Special Publ. 29.

**30.** Nelson JS. Fishes of the World. Fourth Edition. John Wiley and Sons, Inc., New York, NY. 2006.

**31.** Kindermann G, Miljkovic N, Ahnelt H, Stevenson DE. The osteology of *Eucyclogobius newberryi* and *Quietula guaymaise* (Teleostei; Gobiidae), two closely related Gobionellines of the East Pacific. Ann Naturhist Mus Wien. 2007;108B:13-56.

**32.** Thacker C. Phylogeny of Gobioidei and placement within Acanthomorpha and a new classification and investigation of diversification and character evolution. Copeia. 2009;(1):93-104.

**33.** Ruber L, Agorreta A. Chapter 1.2 Molecular Systematics of Gobioid Fishes. Pp. 23-50. In: Patzner RA, Van Tassell JL, Kovacˇic´ M, Kapoor BG, editors. The biology of gobies. CRC Press, Boca Raton, FL. 2011.

**34.** Pezold F. Chapter 1.6. Systematics of Gobionellidae. Pp. 87-97. In: Patzner RA, Van Tassell JL, Kovacˇic´ N, Kapoor BG, editors. The biology of gobies. CRC Press, Boca Raton, FL. 2011.

**35.** Van Tassell JL. Chapter 2.1 . Gobiiformes of the Americas. Pp. 139-176. In: Patzner RA, Van Tassell JL, Kovacˇic´ M, Kapoor BG, editors. The biology of gobies. CRC Press, Boca Raton, FL. 2011.

**36.**  Thacker C. Phylogenetic placement of the European sand gobies in Gobionellidae and characterization of gobionellid lineages (Gobiiformes: Gobioidei). Zootaxa. 2013;3619(3):369-382.

**37.** Tornabene L, Chen Y, Pezold F. Gobies are deeply divided: phylogenetic evidence from nuclear DNA. Syst Biodiv. 2013;1-17.

**38.** Agorreta A, San MD, Schliewen U, Van Tassell JL, Kovacˇic´ M, Zardoya R, et al. Molecular phylogenetics of Gobioidei and phylogenetic placement of European gobies. Mol Phylo Evol. 2013;69(2):619-633.

**39.** Ellingson RA, Swift CC, Findley LT, Jacobs DK. Convergent evolution of morphological adaptations in geographically isolated Bay gobies (Teleostei: Gobionellidae) of the temperate North [Pacific. Mol Phylogenet Evol. 2014;70(2):464-477. http://dx.doi.org/10.1016/j.ympev.2013.10.009.](Pacific.%20Mol%20Phylogenet%20Evol.%202014;70(2):464-477.%20http://dx.doi.org/10.1016/j.ympev.2013.10.009.)

**40.** Girard C. A list of the fishes collected in California by Mr. E. Samuels, with descriptions of new species. Boston J Nat Hist. 1857;6:533-541.

**41.** Girard C. Fishes of North America, observed on a survey for a railroad route from the Mississippi River to the Pacific Ocean. Washington, D. C. 1858,1859 (Two identical editions).

**42.** Gunther A. Catalogue of the fishes in the British Museum. Catalogue of the acanthopterygian fishes in the collection of the British Museum. Gobiidae…[thru]…Notacanthi. Trustees of the British Museum, London, UK, 1861.

**43.** Steindachner F. Ichthyologische beitrage VIII. Sitzungsberichte der akademie der Wissenschaften. 1879;80:119-191.

**44.** Gill TN. Prodromus descriptionis subfamiliae Gobinarum squamis cycloideis piscium, cl W. Stimpsono in mare Pacifico acquisitorum. Ann Lyceum Nat Hist, New York. 1859;6(1-3):12-16.

**45.** Lockington WN. Report upon the edible fishes of the Pacific coast, U.S.A. Pp. 16-66. In: Report of the Commissioners of Fisheries of the State of California for the year 1880. 1880.

**46.** Jordan DS, Gilbert CH. List of the fishes of the Pacific coast of the United States, with a table showing the distribution of the species. Proc US Nat Mus. 1881;3(173):452-458.

**47.** Eigenmann CH, Eigenmann RS. A catalogue of the fishes of the Pacific coast of America north of Cerros Island. Ann New York Acad Sci. 1892;6(Art. 6):349-358.

**48.** Ginsburg I. Contribution to a methodology in the caudal fin ray count in fishes and its use in classification. Copeia. 1945;(3)133-142.

**49.** Jordan DS. Notes on the freshwater fishes of San Luis Obispo County, California. Bull US Fish Comm. 1895;14:141-142.

**50.** Hubbs CL. Description of a new genus and species of goby form California with notes on related species. Occ Pap Mus Zool Univ Mich. 1921;(99):1-5.

**51.** Hubbs CL. Notes on the gobioid fishes of California, with descriptions of two new genera. Occ Pap Mus Zool Univ Mich. 1926;(169):1-6.

**52.** Ulrey AB, Greeley PO. A list of marine fishes (Teleostei) of southern California with their distribution. Bull So Calif Acad Sci. 1928;26(1):1-53.

**53.** Ulrey AB. A check-list of the fishes of southern California and lower California. J Pan-Pac Res Inst. 1929;4(4):2-11.

**54.** Evermann BW, Clark HW. A distributional list of the species of freshwater fishes known to occur in California. Calif Dept Fish Game Fish Bull. 1931;35:1-67.

**55.** Barnhart PS. Marine fishes of Southern California. University of California Press, Berkeley, CA. 1936.

**56.** Schrenkeisen R. Field book of fresh-water fishes of North America north of Mexico. G. P. Putnams’s Sons, New York, NY. 1938.

**57.** Dill WA, Shapovalov L. California freshwater fishes and their possible use for aquarium purposes. Calif Fish Game. 1939;25(4):313-324.

**58.** Needham PR. Quantitative and qualitative observations on fish foods in Waddell Creek lagoon. Trans Amer Fish Soc. 1940;69(2):178-186.

**59.** Shapovalov L. The fresh-water fish fauna of California. Proc Sixth Pac Sci Cong. 1943;3:441-446.

**60.** Hubbs C. Mixture of marine and freshwater fishes in the lower Salinas River, California. Copeia. 1947;(2):147-149.

**61.** Shapovalov L, Dill WA. A checklist of the fresh-water and anadromous fishes of California. Calif Fish Game. 1950;36(4):382-391.

**62.** Shapovalov L, Taft AC. The life histories of the steelhead rainbow trout (*Salmo gairdnerii gairdnerii*) and silver salmon (*Oncorhynchus kisutch*) with special reference to Waddell Creek, California. Calif Dept Fish Game Fish Bull. 1954;98: pp. 375.

**63.** Eddy S. How to know the freshwater fishes. William C. Brown Co., Dubuque, Iowa. 1957.

**64.** Shapovalov L, Dill WA, Cordone AJ. A revised checklist of the freshwater and anadromous fishes of California. Calif Fish Game. 1959;45(3):159-180.

**65.** Kimsey JB, Fisk LO. Keys to the freshwater and anadromous fishes of California. Calif Fish Game. 1960;46(4):453-479.

**66.** Kimsey JB, Fisk LO. Freshwater nongame fishes of California. Calif Fish Game, Sacramento, CA. 1964;1-54.

**67.** Bailey RM, Lachner EA, Lindsey CC, Robins CR, Roedel PM, Scott WB, Woods LP. A list of common and scientific names of fishes from the United States and Canada. Second Edition. American Fisheries Society, Ann Arbor, MI Special Publication No. 2, Ann Arbor, MI. 1960.

**68**. Eddy S. How to know the Freshwater Fishes, 2^nd^ ed. William C. Brown Co., Dubuque, Iowa. 1969.

**69.** Hubbs CL. Miller RR. Studies of cyprinodont fishes. XXII. Variation in *Lucania parva*, its establishment in western United States, and descriptions of a new species from an interior basin in Coahuila, Mexico. Misc Publ Mus Zool, Univ Mich. 1965;127:1-104.

**70.** Bailey RM, Fitch JE, Herald ES, Lachner EA, Lindsey CC, Robins CR, et al. A list of the common and scientific names of fishes from the United States and Canada. Third Edition. American Fisheries Society, Washington, D. C. Special Publication No. 6. 1970.

**71.** Miller DJ, Lea RN. Guide to the coastal Marine fishes of California. Calif Dept Fish Game Fish Bull. 1972;157:1-235.

**72.** Eldridge MB, Bryan CF. Larval fish survey of Humboldt Bay, California. Nat Mar Fish Ser, Tech Rep. 1972;SSRF-665:1-8.

**73.** Fierstine HL, Kline KF, Garman GR. Fishes collected in Morro Bay, California between January, 1968 and December, 1970. Calif Fish Game.1973;59( 1):73-88.

**74.** Miller DJ, Lea RN. Addendum. In: Guide to the coastal marine fishes of California. Calif Dept Fish Game Fish Bull. 1976;157:237-249. (Reprint).

**75.** Moyle PB. Inland Fishes of California. University of California Press, Berkeley, CA. 1976.

**76.** Shiino SM. List of common names of fishes of the world. Those prevailing among English-speaking nations. Sci Rept Shima Marineland. 1976;4:1-262.

**77.** Eddy S, Underhill JC. How to Know the Freshwater Fishes. William C. Brown Co., Dubuque, Iowa. 1978.

**78.** Deacon JE, Kobetich G, Williams JD, Contreras S, et al. Fishes of North America endangered, threatened, or of special concern 1979. Fisheries. 1978;4(2):30-44.

**79.** Hubbs CL, Follett WI, Demster LJ. List of the fishes of California. Occ Pap Calif Acad Sci. 1979;133:1-51.

**80.** Swift CC. *Eucyclogobius newberryi* (Girard). In: Lee DS et al. Atlas of North American Freshwater Fishes. North Carolina State Museum, Raleigh, NC. 1980; pp. 788.

**81.** Robins CR, Bailey RM, Bond CE, Brooker JR, Lachner EA, Lea RN, et al. A list of common and scientific names of fishes from the United States and Canada. Fourth Edition. American Fisheries Society, Bethesda, MD, Special Publication No. 12. 1980.

**82.** Gotshall, DW, Allen GH, Barnhart RA. An annotated checklist of fishes from Humboldt Bay, California. Calif Fish Game. 1980;66(2):220-232.

**83.** Ono RD, Williams JD, Wagner A. Vanishing Fishes of North America. Stone Wall Press, Inc. Washington, D.C. 1983.

**84.** Eschmeyer WN, Herald ES, Hamann H. A field guide to Pacific coast fishes of North America. Houghton Mifflin Co., Boston, MA. 1983.

**85.** Leidy RA. Distribution and ecology of stream fishes in the San Francisco Bay drainage. Hilgardia. 1984;52(8): pp. 175.

**86.** McGinnis SM. Freshwater fishes of California. California Natural History Guides 49, University of California Press, Berkeley, CA. 1984.

**87.** Minckley WL, Hendrickson DA, Bond CE. Geography of western North American freshwater fishes: description and relationships to intracontinental tectonism. Pp. 519-613. In: Hocutt CH, Wiley EO, editors. The Zoogeography of North American Freshwater Fishes. John Wiley and Sons, New York, NY. 1986.

**88.** Swift CC, Nelson JL, Maslow C, Stein T. 1989. Biology and distribution of the Tidewater Goby, *Eucyclogobius newberryi* (Pisces: Gobiidae) of California. Contrib Sci Nat Hist Mus, Los Angeles, CA. 1989;404:1-19.

**89.** Williams JE, Johnson JE, Hendrickson DA, Contreras-Balderas S, Williams JD, Navarro-Mendoza M, et al. Fishes of North America endangered, threatened, or of special concern. Fisheries. 1989;14(6):2-20.

**90.** Moyle PB, Williams JE. Biodiversity loss in the temperate zone: Decline of the native fish fauna of California. Cons Biol. 1990;4(1):275-284.

**91.** Swift CC. Early California wetlands. Tidelines. 1990;10(1):4-5.

**92.** Robins CR, Bailey RM, Bond CE, Brooker JR, Lachner EA, Lea RN, et al. A list of common and scientific names of fishes from the United States and Canada. Fifth Edition. American Fisheries Society, Bethesda, MD. Special Publication No. 20. 1991.

**93.** Page LM, Burr BM. A Field Guide to freshwater fishes. North America north of Mexico. Houghton Mifflin Company, New York, NY. 1991.

**94.** Yoklavich MM, Cailliet GM, Barry JP, Ambrose DA, Antrim BS. Temporal and spatial patterns in abundance and diversity of fish assemblages in Elkhorn Slough, California. Estuaries. 1991;14(4):465-480.

**95.** Mayden RL, Burr BM, Page LM, Miller RR. The native freshwater fishes of North America. Pp. 825-863. In: Mayden RL, editor. Systematics, historical ecology, and North American freshwater fishes. Stanford University Press, Stanford, CA. 1992.

**96.** Monaco ME, Lowery TA, Emmett RL. Assemblages of U. S. west coast estuaries based on the distribution of fishes. J Biogeog. 1992;19(1):251-267.

**97.** Chamberlain RH, Barnhart RA. Early use by fish of a mitigation salt marsh, Humboldt Bay, California. Estuaries. 1993;16(4):769-783.

**98.** Swenson RO. Marshes build better gobies: Population dynamics and reproduction in an estuarine fish, *Eucyclogobius newberryi.* Amer Zool. 1993;33:441.

**99.** Swift CC, Haglund TR, Ruiz M, Fisher RN. The status and distribution of the freshwater fishes of southern California. Bull So Calif Acad Sci. 1993;92(3):101-167.

**100.** Brown C, Swenson R. Tidewater goby. In: Thelander CG, Crabtree M, editors. Life on the edge. A guide to California’s endangered natural resources. Biosystems Analysis, Inc., CA. 1994; pp. 390-391.

**101.** Swenson RO, McCray AT. Feeding ecology of the Tidewater Goby. Transactions of the American Fisheries Society. 1996;125(4):956-970.

**102.** Watson, W. Gobiidae. Gobies. Atlas 33, pp. 1214-1245. In: H. Geoffrey Moser, Editor. The early stages of fishes in the California Current Region. Calif Coop Ocean Fish Invest. 1996..

**103.** Lafferty KD, Swenson RO, Swift CC. Threatened fishes of the world: *Eucyclogobius newberryi* Girard, 1857 (Gobiidae). Environ Biol Fish. 1996;46(2):254.

**104.** Love M. Probably more than you want to know about the fishes of the Pacific Coast. 2^nd^ Edition. Really Big Press, Santa Barbara, CA. 1996.

**105.** Swenson RO. Sex-role reversal in the Tidewater Goby, *Eucyclogobius newberryi*. Environ Biol Fish. 1997;50(1):27-40.

**106.** Capelli MH. Tidewater goby (*Eucyclogobius newberryi*) management in California estuaries. Proc Calif World Ocean Conf, San Diego, CA. 1997; pp. 1-17.

**107.** Lafferty KD, Page CJ. Predation on the endangered Tidewater Goby, *Eucyclogobius newberryi*, by the introduced African clawed frog, *Xenopus laevis,* with notes on the frog’s parasites. Copeia. 1997;(3):589-592.

**108.** Lafferty KD, Swift CC, Ambrose RF. Post-flood persistence and recolonization of endangered Tidewater Goby populations. N Amer J Fish Manag. 1999a;19(2):618-622.

**109.** Lafferty KD, Swift CC, Ambrose RF. Extirpation and recolonization in a metapopulation of an endangered fish, the Tidewater Goby. Cons Biol. 1999b;13(6):1447-1453.

**110.** Swenson RO. The ecology, behavior, and conservation of the Tidewater Goby, *Eucyclogobius newberryi*. Environ Biol Fish. 1999;55(1):99-119.

**111.** Fuller PL, Nico LG, Williams JD. Nonindigenous fishes introduced into inland waters of the United States. Special Publication No. 27, American Fisheries Society, Bethesda, MD. 1999.

**112.** Ambrose RF and Meffert DJ. Fish-assemblage dynamics in Malibu Lagoon, a small, hydrologically altered estuary in Southern California. Wetlands. 1999;19(2):327-340.

**113.** Musick JA, Harbin MM, Berkeley SA, Burgess GA, Eklund AM, Findley L et al. Marine, estuarine, and diadromous fish stocks at risk of extinction in North America (exclusive of Pacific salmonids). Fisheries. 2000;25(11):6-30.

**114.** Moyle PB, David LH. A list of freshwater, anadromous, and euryhaline fishes of California. Calif Fish Game. 2000;86(4):244-258.

**115.** Matern SA. Using temperature and salinity tolerances to predict the success of the shimofuri goby, a recent invader into California. Trans Amer Fish Soc. 2001;130(2):592-599.

**116.** Mendoca H, Smith J, Brinegar C. Isolation and characterization of four microsatellite loci in the Tidewater Goby (*Eucyclogobius newberryi*). Mar Biotech. 2001;3(1):91-95.

**117.** Dawson MN, Stanton JL, Jacobs DK. Phylogeography of the Tidewater Goby, *Eucyclogobius newberryi* (Teleostei, Gobiidae), in coastal California. Evol. 2001;55(6):1167-1179.

**118.** Swift CC, Swenson RO, Hieb K. Gobies. Pp. 7-9. In: Leet WS, Dewees CM, Klingbeil R, Larson EJ, editors, California Living Resources: A status report. The Errata. Calif Dept Fish Game, Sacramento, CA. 2002. (printed 2001).

**119.** Moyle PB. Inland Fishes of California. Revised and Expanded. University of California Press, Berkeley, CA. 2002.

**120.** Dawson MN, Louie KD, Barlow M, Jacobs DK, Swift CC. Comparative phylogeography of sympatric sister species, *Clevelandia ios* and *Eucyclogobius newberryi* (Teleostei, (Gobiidae), across the California transition zone. Mol Ecol. 2002;11(3):1065-1075.

**121.** Moeller A, MacNeil SD, Ambrose RF, Que Hee SS. Elements in fish of Malibu Creek and Malibu Lagoon near Los Angeles, California. Marine Poll Bull. 2003;46:424-429.

**122.** Anhelt H, Goschl J, Dawson MN, Jacobs DK. Geographical variation in the cephalic lateral line canals of *Eucyclogobius newberryi* (Teleostei, Gobiidae) and its comparison with molecular phylogeography. Folia Zool. 2004;53(4):385-398.

**123.** Marchetti MP, Light T, Moyle PB, Viers JH. Fish invasions in California watersheds: Testing hypotheses using landscape patterns. Ecol Appl. 2004;14(5):1507-1525.

**124.** Jacobs DK, Haney TA, Louie KD. Genes, diversity, and geological processes on the Pacific Coast. Ann Rev Earth Planet Sci. 2004;32:601-652.

**125.** U.S. Fish and Wildlife Service. Recovery Plan for the Tidewater Goby (*Eucyclogobius newberryi*). U.S. Fish and Wildlife Service, Pacific Region, Portland, OR. 2005.

**126.** Leidy RA, Becker G, Harvey BN. Historical status of coho salmon in streams of the urbanized San Francisco estuary, California. Calif Fish Game. 2005;91(4):219-254.

**127.** Swift CC. Chapter 29. The Distribution of Fishes. Pp. 601-638. In: Barton M, Bond’s Biology of Fishes. Third Edition. Thompson Brooks/Cole, Belmont, CA. 2006.

**128.** Barton M. Bond’s Biology of Fishes. Third Edition. Thompson Brooks/Cole, Belmont, CA. 2006.

**129.** McGinnis SM. Field Guide to freshwater fishes of California. Revised Edition. California Natural History Guides Series 77, University of California Press, Berkeley, CA. 2006.

**130.** Horn MH, Allen LG, Lea RN. Chapter 1. Biogeography. Pp. 3-25. In: Allen LG, Pondella, DJ II, Horn MH, editors. The ecology of marine fishes. California and Adjacent Waters. University of California Press, Berkeley, CA. 2006.

**131.** Dawson MN, Waples RS, Bernardi G. Chapter 2. Phylogeography. Pp. 26-54. In: Allen LG, Pondella DJ II, Horn MH, editors. The ecology of marine fishes. California and Adjacent Waters. University of California Press, Berkeley, CA. 2006.

**132.** Allen LG, Yoklavich MM, Cailliet GM, Horn MH. Chapter 5. Pp. 119-148. Bays and estuaries. In: Allen LG, Pondella DJ II, Horn MH, editors. The ecology of marine fishes. California and Adjacent Waters. University of California Press, Berkeley, CA. 2006.

**133.** Chamberlain RH. Environmental variables of northern California lagoons and estuaries and the distribution of Tidewater Goby (*Eucyclogobius newberryi*). U.S. Fish and Wild Serv, Arcata Fisheries Tech Rep, TR 2006-04, Arcata, California. 2006.

**134.** McGourty KR, Kinziger AP, Henrickson GL, Goldsmith GH, Casal G, Azevedo C. A new microsporidian infecting the musculature of the endangered Tidewater Goby (Gobiidae). J Parasit. 2007;93(3):655-660.

**135.** McGourty KR, Kinziger AP, Goldsmith GH. Spawning time, fecundity, habitat utilization, and parasites of a northern California population of Tidewater Goby, *Eucyclogobius newberryi*. Calif Fish Game. 2008;94(1):18-32.

**136.** Frimodig AJ, Goldsmith GH. First record of a cymothoid isopod from a Tidewater Goby and three new Tidewater Goby localities in Humboldt County, California. Calif Fish Game. 2008; 94(4):194-199.

**137.** Jelks HL, Walsh SJ, Burkhead NM, Contreras-Balderas S, Diaz-Pardo E, Hendrickson DA, et al. Conservation Status of Imperiled North American Freshwater and diadromous fishes. Fisheries. 2008;33(8):372-407.

**138.** Earl DA, Louie KD, Bardeleben C, Swift CC, Jacobs DK. Rangewide microsatellite phylogeography of the endangered Tidewater Goby, *Eucyclogobius newberryi* (Teleostei: Gobiidae), a genetically subdivided coastal fish with limited marine dispersal. Cons Genet. 2010. DOI:10.1007/s10592-009-0008-9.

**139.** McCraney WT, Goldsmith GH, Jacobs DK, Kinziger AP. Rampant drift in artificially fragmented populations of the endangered Tidewater Goby (*Eucyclogobius newberryi*). Mol Ecol. 2011;19(16):3315-3327.

**140.** Love MS. Certainly more than you want to know about the fishes of the Pacific Coast. A post modern experience. Really Big Press, Santa Barbara, CA. 2011.

**141.** Borges R, Faria C, Gil F, Goncalves EJ. Chapter 3.4. Early Development of Gobies. Pp. 403-462. In: Patzner RA, Van Tassell JL, Kovacic M, Kapoor BG, editors. The biology of gobies. CRC Press, Boca Raton, FL. 2011.

**142.** U.S. Fish and Wildlife Service. Endangered and Threatened Wildlife and Plants; Final Designation of critical habitat for the Tidewater Goby. Federal Register. 2013;78(25):8746-8819.

**143.** Page LM, Espinosa-Perez H, Findley LT, Gilbert CR, Lea RN, Mandrak NE, et al. Common and scientific names of Fishes from the United States, Canada, and Mexico. 7^th^ Edition. American Fisheries Society, Bethesda, MD. Special Publication 34. 2013.

**144.** Dawson MN. Natural experiments and meta-analyses in comparative phylogeography. J Biogeog. 2014;41(1):52-65.

**145.** Spies BT, Tarango BC, Steele MA. Larval duration, settlement, and larval growth rates of the endangered Tidewater Goby (*Eucyclogobius newberryi*) and the Arrow Goby (*Clevelandia ios*) (Pisces, Teleostei). Bull So Calif Acad Sci. 2015;113(3):165-175 (printed 2014).

**146.** Richmond JQ, Jacobs DK, Backlin AR, Swift CC, Dellith C, Fisher RN. Ephemeral stream reaches preserve the evolutionary and distributional history of threespine stickleback in the Santa Clara and Ventura river watersheds of southern California. Conserv Genet. 2014;16:85-101. DOI:10.007/s10592-014-0643-7.

**147.** Hellmair M, Kinziger AP. Increased extinction potential of insular fish populations with reduced life history variation and low genetic diversity. PLoS One. 2014;9(11):e113139. DOI:101371/journal.pone.0113139.

**148.** Collins DG, Melack JM. Biological and chemical responses in a temporarily open/closed estuary to variable freshwater inputs. Hydrobiologia. 2014;734:97-113. DOI:10.1007/s10750-014-1872-y.

**149.** Martin KL. Theme and variations: amphibious air-breathing intertidal fishes. J Fish Biol. 2014;84:577-602. DOI:10.1111/jfb.12270.

**150.** Swift CC, Howard S, Mulder J, Pondella DJ II, Keegan TP. Expansion of the non-native Mississippi silverside, *Menidia audens* (Pisces, Atherinopsidae), into fresh and marine waters of coastal southern California. Bull So Calif Acad Sci. 2015;113(3):153-164 (printed 2014).

**151.** Kinziger AP, Hellmair M, McCraney T, Jacobs DK, Goldsmith G. Temporal genetic analysis of the endangered tidewater goby: Extinction-colonization dynamics or drift in isolation? Mol Ecol. 2015;24(22):1-10. DOI:10.1111/mec.13424.

**152.** Martin KL. Beach spawning fishes: Reproduction in an endangered ecosystem. CRC Press, Boca Raton, FL. 2015.

**153.** Chase DA, Todgham AE. Effects of species assemblage on juvenile growth and condition in three California estuarine fishes. Trans Amer Fish Soc. 2016;145(1):94-107. <http://dx.doiorg/10.1080/00028487.2015.1106420>.

**154.** Schmelzle MC, Kinziger AP. Using occupancy modeling to compare environmental DNA to traditional field methods for regional-scale monitoring of an endangered aquatic species. Mol Ecol Resour. 2016. DOI:10.1111/17550998.12501.

**155.** Spies BT, Steele MA. Effects of temperature and latitude on larval traits of two estuarine fishes in differing estuary types. Mar Ecol Prog Ser. 2016;544:243-255. DOI:10.3354/meps11552.

**156.** Starks EC, Morris EL. The marine fishes of southern California. Univ Calif Publ Zool. 1907;3(11):159-251.

**157.** Snyder CO. A study of the trout (*Salmo irideus* Gibbons) from Waddell Creek, California. Calif Fish Game. 1938;24(4):354-375.

**158.** Metz CW. The fishes of Laguna Beach, California. Ann Rept Laguna Mar Lab. 1912;1:19-66.

**159.** Miller RR. Occurrence of the cyprinodont fish *Fundulus parvipinnis* in fresh water in San Juan Creek, southern California. Copeia. 1939;(3):168.

**160.** Miller RR. Further data on freshwater populations of the Pacific killifish, *Fundulus parvipinnis*. Copeia. 1943;(1):41-42.

**161.** Goldberg SR. Seasonal ovarian cycle of the Tidewater Goby, *Eucyclogobius newberryi* (Gobiidae). Southwest Nat. 1977;22(4):557-559.
